# Consequences of Social Distancing Measures During the COVID-19 Pandemic First Wave on the Epidemiology of Children Admitted to Pediatric Emergency Departments and Pediatric Intensive Care Units: A Systematic Review

**DOI:** 10.3389/fped.2022.874045

**Published:** 2022-06-03

**Authors:** Michael Levy, Victor Lestrade, Carla Said, Philippe Jouvet, Atsushi Kawaguchi

**Affiliations:** ^1^Department of Pediatrics, Centre Hospitalier Universitaire Sainte-Justine, Pediatric Critical Care, University of Montreal, Montréal, QC, Canada; ^2^Pediatric Intensive Care Unit, Centre Hospitalier Universitaire Robert-Debré, Assistance Publique Hôpitaux de Paris, Université de Paris, Paris, France; ^3^School of Medicine, University of Paris Saclay, Paris, France; ^4^Department of Intensive Care Medicine, Pediatric Critical Care Medicine, Tokyo Women's Medical University, Tokyo, Japan

**Keywords:** social distancing measures, school closure, lockdown, pediatrics, emergency care, pediatric intensive care unit (PICU), SARS-CoV-2, pandemic (COVID-19)

## Abstract

**Objectives:**

To synthesize knowledge describing the impact of social distancing measures (SDM) during the first wave of the COVID-19 pandemic on acute illness in children by focusing on the admission to pediatric emergency departments (PED) and pediatric intensive care units (PICU).

**Methods:**

We searched Cochrane Database of Systematic Reviews, Cochrane Controlled Trials Register, EPOC Register, MEDLINE, Evidence-Based Medicine Reviews, EMBASE, WHO database on COVID-19, Cochrane Resources on COVID-19, Oxford COVID-19 Evidence Service, Google Scholar for literature on COVID-19 including pre-print engines such as medRxiv, bioRxiv, Litcovid and SSRN for unpublished studies on COVID-19 in December 2020. We did not apply study design filtering. The primary outcomes of interest were the global incidence of admission to PICU and PED, disease etiologies, and elective/emergency surgeries, compared to the historical cohort in each studied region, country, or hospital.

**Results:**

We identified 6,660 records and eighty-seven articles met our inclusion criteria. All the studies were with before and after study design compared with the historical data, with an overall high risk of bias. The median daily PED admissions decreased to 65% in 39 included studies and a 54% reduction in PICU admission in eight studies. A significant decline was reported in acute respiratory illness and LRTI in five studies with a median decrease of 63%. We did not find a consistent trend in the incidence of poisoning, but there was an increasing trend in burns, DKA, and a downward trend in trauma and unplanned surgeries.

**Conclusions:**

SDMs in the first wave of the COVID-19 pandemic reduced the global incidence of pediatric acute illnesses. However, some disease groups, such as burns and DKA, showed a tendency to increase and its severity of illness at hospital presentation. Continual effort and research into the subject should be essential for us to better understand the effects of this new phenomenon of SDMs to protect the well-being of children.

**Systematic Review Registration:**

Clinicaltrials.gov, identifier: CRD42020221215.

## Introduction

Severe Acute Respiratory Syndrome Coronavirus-2 (SARS-Cov-2) and its pandemic have severely affected the healthcare system worldwide as well as our social lives. Studies have demonstrated that children with COVID-19 are responding differently from the elderly population with less severity of illness requiring less emergency and critical care resources (1–4). Preventing transmission particularly to the elderly population and slowing the rate of infections in the community to maintain the healthcare system resulted mainly in social distancing measures (SDMs) including school closures (SCs), which came from the experience of the influenza epidemic and other previous pandemics, particularly during the first wave of this pandemic (5–7). Hospitals have also taken specific measures to decrease their activity in the prevision of an increase in COVID-19 admissions and workforce shortage (8). Both SDMs and hospital measures have come with many trade-offs that have impacted acute illness of children requiring Pediatric Emergency Department (PED) or Pediatric Intensive Care Unit (PICU) admission. After the first wave, various countries and regions have reported their results and insights into the consequences of their respective SDMs. This review aims to synthesize knowledge describing the impact of the SDMs together with other various measures of the first wave of the pandemic, defined in each country by the pandemic outbreak until the specific peak and secondary decline, on acute illness requiring hospital admission in children by focusing on PED and PICU admissions as well as on the main type of acute illness or injury in children.

## Methods

In this review, we followed the methodology for data collection and analysis in the Cochrane Handbook for Systematic Reviews of Interventions (9). The study protocol was registered in the PROSPERO database (registration number: CRD42020221215).

### Eligibility Criteria

#### Participants

We included pediatric patients including neonates, infants and children/adolescents regardless of gender, countries, regions, and ethnic groups.

#### Intervention

The impacts of the “pandemic” itself, and other medical and social measures such as social distancing measures (e.g., school closure or mandatory mask-wearing) and hospital-based clinical measures such as postponing planned surgery or restricting the planned surgery related to the COVID-19 pandemic first wave in 2020.

#### Comparators

The historical cohort in each studied region, country, or hospital.

#### Outcomes

The global incidence of admission to PED and PICU.

- Incidence of PED admission of children with i) acute respiratory illness including respiratory illness, bronchiolitis and asthma, ii) viral infections, iii) injuries including trauma, burns and poisoning, iii) diabetic ketoacidosis (DKA), and iiii) unplanned surgery.
- Incidence of elective/emergency surgeries like neurosurgery and cardiac surgery.
- These categories of illness were chosen according to the usual epidemiology of children admitted to PED and to PICU and included diseases that had consensual definitions.

### Search Sources

Studies were identified from journal literature and conference proceedings via systematic searches of bibliographic databases including the Cochrane Library (Cochrane Database of Systematic Reviews, Cochrane Controlled Trials Register and the EPOC Register), MEDLINE, Evidence-Based Medicine Reviews (OVID) and Embase. On top of those databases, WHO database on COVID-19, Cochrane Resources on COVID-19, Oxford COVID-19 Evidence Service, Google Scholar for literature on COVID-19 including pre-print engines such as medRxiv, bioRxiv, Litcovid and SSRN for unpublished studies on COVID-19 were searched. Electronic databases were explored for all studies published articles before December 8, 2020, using the search terms addressed in Appendix 1.

### Study Selection

To be included in the review, studies needed to compare the number of admissions for the main selected diseases within two time periods: during the COVID-19 pandemic first wave period of SDM, as compared to the previous year(s), or to the period just before. Only articles in the English language were included.

Following the removal of duplicate studies, two authors (AK and ML) independently assessed the eligibility of the studies using Rayyan (https://rayyan.qcri.org/welcome). Studies were selected as being potentially relevant by screening the titles and abstracts. A third reviewer (VL) assessed all the titles and abstracts that remained in disagreement between the two investigators. We obtained the full text of the article for review when a decision cannot be made by screening the title and the abstract. The two review authors retrieved the full texts of all potentially relevant articles and independently assessed their eligibility by filling out eligibility forms designed by the specified inclusion criteria.

### Data Extraction and Quality Assessment

Data from the eligible studies were extracted by two investigators using a prespecified data extraction form to record demographic data (including age, country/region), study details (aims/study question, country of origin, methods/study designs), and study outcomes.

Study quality and risk of bias was undertaken according to the Johanna Briggs Institute appraisal tool for analytical cross-sectionals studies (10). However, we did not include the assessment results of the study quality and risk of bias for each included study because all the corresponding studies were with before and after the design of the study with historical cohorts, indicating a high risk of bias without exceptions.

### Data Syntheses and Analyses

Data regarding incidence in all the included studies were expressed in mean daily admissions during each study period (Period of social distancing measures = SDM period versus control period). The comparison was made using the percentage of change between the SDM period and the control periods. Median differences and interquartile ranges [IQR] were calculated for each outcome. For all the variables expressed in absolute numbers and percentages (i.e., hospital admission following PED attendance, very urgent triage codes (including patients that either required immediate resuscitation or had life-threatening conditions that required prompt evaluation by the emergency team) and PICU admissions), effects of the study period on these outcomes were reported using odds ratios (OR) and 95% confidence interval (CI) estimates.

## Results

**Figure 1:**
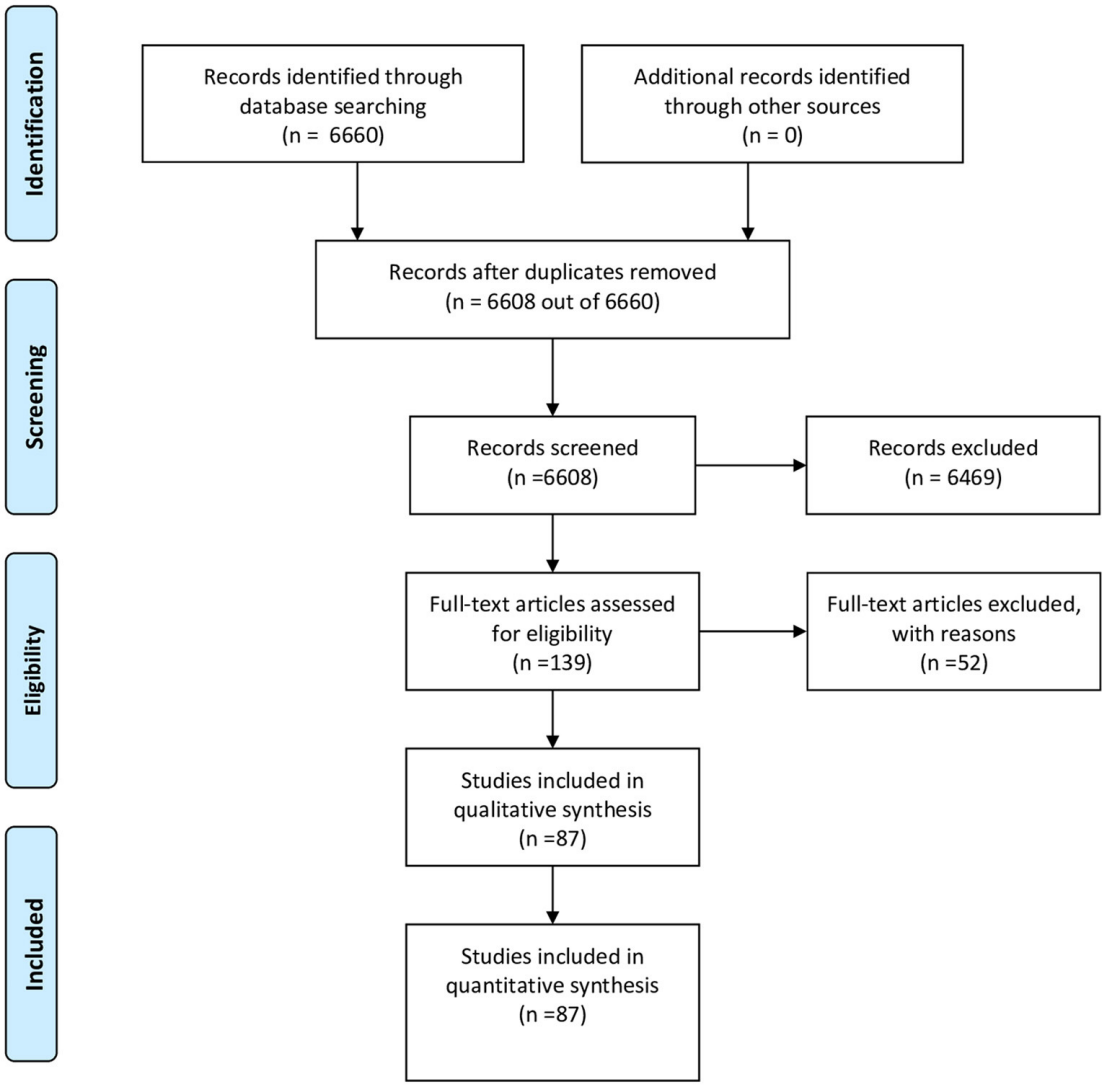
Study Flow Diagram.

We identified 6,660 records through a systematic database search (Figure 1). We removed 52 duplicates, and the titles and abstracts of the remaining 6,608 records were screened for eligibility. We identified 139 potentially eligible articles for the full-text review. After independent assessment by the two investigators, 87 articles met our inclusion criteria and presented actionable data. All the studies were included in this review and were with before and after design with historical cohorts as expected, indicating a high risk of bias without exceptions.

### Countries and Regions

Of the 87 included studies (Table 1), three were international studies. Reports from 26 different countries were identified. To be specific, 15 (17%) were, respectively from the USA and Italy, 13 (15%) from the UK, 4 (5%) from Australia, 3 (3%), respectively from China, France, Ireland, and Spain, 2 (2%), respectively from Brazil, Finland, Germany, India, Japan, New-Zealand, and Turkey. The remaining studies were from Argentina, Cameroon, Canada, Indonesia, Iran, Israel, Malta, Morocco, Scotland, Slovenia, and South Africa (*n* = 1 in each country).

**Table 1:** Included studies.

**References**	Countries (level of income)*	**Multicenter study**	**Number of patients included**	**Age of cohort**	**Settings**	**Type of admission**	**Method used**	**SDM period**	**Comparison period**
Akuaake et al. (11)	South Africa (MIC)	No	9,982	Children <13 yrs.	ED	ED	Before-after	March 27 to April 30, 2020	February 21 to March 26, 2020
									March 27 to April 30, 2019
									March 27 to April 30, 2018
Angoulvant et al. (12)	France (HIC)	Yes	871,543	Children	ED *n* = 6	ED	Time-series analysis	March 17 to April 19, 2020	February 15 to March 16, 2020
Araujo et al. (13)	Brazil (MIC)	Yes	8,654	Children	PICU *n* = 15	PICU, bronchiolitis, asthma	Interrupted time-series method	March 1 to May 31, 2020 March 1 to May 31, 2020	March 1 to May 31, 2017-19
Bastemur E	UK (HIC)	No	136	Children	transportation team	DKA	Before after	March 1 to July 31, 2020	March 1 to July 31, 2015-19
Baxter et al. (14)	UK (HIC)	No	329	Children	ED	Trauma	Before after	March 24 to May 10, 2020	March 24, 2020, to May 10, 2019
Bram et al. (15)	USA (HIC)	No	1,745	Children	ED + Clinic + virtual	Trauma	Before after	March 15 to April 15, 2020	March 15 to April 15, 2018-19
Bressan et al. (16)	Italy (HIC)	No	3,713	Children > 1 yr.	ED	Trauma, Burn, Poisoning	Before after	March 8 to April 20, 2020	March 8 to April 20, 2019
Britton et al. (17)	Australia (HIC)	No	NA	Children	ED	Bronchiolitis, RSV	Time-series analysis	April 1 to June 30, 2020	Jan 1, 2015, to March 30, 2020
Chavasse et al. (17)	UK (HIC)	No	NA	Children 1–17 yrs.	ED	Asthma	Before after	Week 12 to 19, 2020	Week 12 to 19 2017-19
									Week 8 to 11, 2020
Chelo et al. (18)	Cameroon (MIC)	No	33,363	Children	HA	ED	Time-series analysis	March 1 to May 31, 2020	March 1 to May 31, 2016–19
Christey et al. (19)	New Zealand (HIC)	No	83	Children <14 yrs. + adults	ED	Trauma	Before after	March 26 to April 8, 2020	March 5 to March 18, 2020
Ciofi degli Atti et al. (20)	Italy (HIC)	No	18,825	Children	ED	ED, Trauma	Before after	March 11 to April 20, 2020	January 1 to February 19, 2020
									February 20 to March 10, 2020
Claudet e tal. (21)	France (HIC)	No	3,137	Children	ED	ED, Trauma	Before after	March 17 to April 19, 2020	March 17 to April 19, 2017-19
Clavenna et al. (22)	Italy (HIC)	No	7,092	Children	ED	ED	Before after	January 1 to March 31, 2020 Feb 23 to March 31, 2020	January 1 to March 31, 2019 January to Feb 23, 2020
								February 24 to March 31, 2020	January 1 to Feb 23, 2020
D'asta F	UK (HIC)	No	10,063	Children	ED	ED, Burn	Before after	March 23 to April 30, 2020	March 23 to April 30, 2019
Dann et al. (23)	Ireland (HIC)	No	21,766	Children	ED	ED, injury and poisoning, surgical, respiratory illnesses	Before after	March 1 to April 30, 2020	March 1 to April 30, 2019
Davico et al.	Italy (HIC)	Yes	15,523	Children <14	ED *n* = 2	ED, seizures	Before after	January 6 to April 19, 2020	January 7 to April 21, 2019
(24)				yrs.				January 6 to February 23, 2020	January 7 to February 24, 2019
								February 24 to April 19, 2020	February 25 to April 21 2019
Dean et al. (25)	USA (HIC)	No	210,358	Children	ED	ED	Time series analysis	December 31, 2019, to May 14, 2020	December 31, to May 14, 2015-19
Degiorgio et al. (26)	Malta (HIC)	No	995	Children <15 yrs.	ED	Medical ED, respiratory illnesses	Before after	March 1 to May 9, 2020	March 1 to May 9, 2019
Dhillon et al. (27)	India (MIC)	No	74	Children 0-12 yrs.	ED	Trauma	Before after	March 25 to May 3, 2020	March 25 to May 3, 2019
								May 4 to May 31, 2020	May 4 to May 31, 2019
Dopfer et al. (28)	Germany (HIC)	No	5,424	Children	ED	ED	Before after	January 1 to April 19, 2020	January 1 to April 19, 2019
Dyson et al. (29)	UK (HIC)	No	146	Children	Neurosurgery admission	Neurosurgery admission	Before after	March 23 to May 3, 2020	March 25 to May 5, 2019
Ferrero et al. (30)	Argentina (MIC)	No		Children 0–18 yrs.	ED	ED	Before after	January 1 to May 31, 2020	January 1 to May 31, 2019
Fisher et al. (31)	USA (HIC)	Yes	1,346	Children	ED n=3	Appendicitis	Before after	March 1 to May 7, 2020	January 1, 2014, to June 1, 2019
Friedrich et al. (32)	Brazil (MIC)	Yes	167,870	Children 0–1 yr.	ED / National database *n* = NA	Bronchiolitis	Times series analysis	March 1 to June 30, 2020	March 1 to June 30, 2016-2019
Gerall et al. (33)	USA (HIC)	No	89	Children	Surgery	Appendicitis	Before after	March 1 to May 31, 2020	March 1, 2019, to May 31, 2019
Giamberti et al. (34)	Italy (HIC)	Yes		Children	Cardiac surgery *n* = 14	Cardiac surgery	Before after	March 9 to May 4, 2020	March 9 to May 4, 2019
Goldman et al. (35)	Canada (HIC)	Yes	88,368	Children 0–16 yrs.	ED *n* = 18	ED	Before after	March 17 to April 30, 2020	March 17 to April 30, 2019
									December 1, 2019, to January 27, 2020
									January 28, 2020, to March 16, 2020
Graciano et al. (24)	USA (HIC)	No	1,487	Children	PICU	PICU, bronchiolitis, asthma	Before after	March 1 to May 31, 2020	March 1 to May 31, 2015-2019
Gunadi et al. (36)	Indonesia (MIC)	No	152	Children	Surgery	Pediatric surgery	Before after	March 1 to May 31 2020	March 1, 2019 – February 29, 2020
Hampton et al. (37)	UK (HIC)	No	5,165	Children + adults	ED	ED, trauma	Before after	March 24 to April 7, 2020	March 24 to April 7, 2019 March 10 to March 23, 2020
Hartnett et al. (38)	USA (HIC)	Yes	>1,600,000	Children 0–14 yrs. + adults	ED *n* = 3552	ED	Before after	March 29 to April 25, 2020	March 31 to April 27, 2019
Hughes et al. (39)	UK (HIC)	Yes	8,915	Children 0-14 yrs. + adults	ED *n* = 109	ED	Before after	March 12 to April 26, 2020	March 14 to April 28, 2019
Iozzi et al. (40)	Italy (HIC)	No	2,956	Children	ED	ED, trauma	Before after	March 10 to May 3, 2020	March 10 to May 3, 2019
Izba et al. (24)	UK/USA (HIC)	Yes	NA	Children 0–16 yrs.	ED *n* = 2	ED	Before after	January 1 to May 20, 2020	January 1 to May, 2019
								Weeks 1–12, 2020	Weeks 1–12, 2019
								Weeks 13–20, 2020	Weeks 13–20, 2019
Kamrath et al. (31)	Germany (HIC)	Yes	1,491	Children	ED *n* = 217	DKA	Before after	March 13 to May 13, 2020	March 13 to May 13, 2018–19
Rose et al. (41)	UK (HIC)	No	4,690	Children 0–16 yrs.	ED	ED, head trauma	Before after	March 21 to April 26, 2020	March 21 to April 26, 2019
Kishimoto et al. (14)	Japan (HIC)	Yes	75,053	Children 0-15 yrs.	HA *n* = 210	Appendicitis, influenza	Interrupted time series analysis	March 1 to June 30, 2020	July 1, 2018, to February 29, 2020
Korun et al. (42)	Turkey (MIC)	No	696	Children	Cardiac surgery	Cardiac surgery	Before after	March 11 to May 11, 2020	March 11, 2019, to March 10, 2020
Krivec et al. (43)	Slovenia (HIC)	No	27	Children	HA	Asthma	Before after	March 16 to April 20, 2020	March 16 to April 20, 2017–19
Kruchevsky et al. (44)	Israel (HIC)	No	8,291	Children + adults	ED	ED, Burn, Trauma	Before after	March 14 to April 20, 2020	March 14 to April 20, 2017–19
Kuitunen et al. (24)	Finland (HIC)	Yes	1,174	Children	ED n=2	ED	Before after	March 16 to April 12, 2020	February 17 to March 15, 2020
Kvasnovsky et al. (45)	USA (HIC)	No	205	Children	Surgery	Appendicitis	Before after	March 31 to May 3, 2020	March 31 to May 3, 2017–2019
Lalarukh et al. (46)	UK (HIC)	No	NA	Children <18 yrs.	ED	ED	Before after	March 1 to May 31, 2020	March 1 to May 31, 2019
Lawrence et al. (47)	Australia (HIC)	No	43	Children <18 yrs.	ED	ED, DKA	Before after	March 1 to May 31, 2020	March 1 to May 31, 2015-19
Lee et al. (48)	USA/Singapore/ Australia/France (HIC)	Yes	NA	Children	ED *n* = 5	ED, PICU	Time series analysis	December 1, 2017, to August 10, 2020	
Lee-Archer et al. (49)	Australia (HIC)	No	105	Children	Surgery	Appendicitis	Before after	March 16 to May 5, 2020	March 16 to May 5, 2019
Lv et al. (24)	China (MIC)	Yes	194	Children + adults	ED *n* = 11	Fractures	Before after	January 20, to February 19, 2020	January 31 to March 2, 2019
Mann et al. (50)	UK (HIC)	No	147	Children	ED	Burns	Before after	March 23 to May 31, 2020	March 23 to May 31, 2019
Manzoni et al. (48)	Italy (HIC)	Yes	1,654	Children	ED *n* = 2	ED, Trauma, Urgent surgery	Before after	March 1 to April 30, 2020	March 1 to April 30, 2019
Matthay et al. (51)	USA (HIC)	No	20,129 C+A	Children <15 yrs. + adults	ED	Trauma	Interrupted time-series analysis	March 17 to June 30, 2020	January 1, 2015, to March 17, 2020
McDonnell et al. (52)	Ireland (HIC)	Yes	61,317	Children	ED *n* = 5	ED	Before after	February 29 to March 12, 2020	February 29 to March 12, 2018–19
								March 13 to March 27, 2020	March 13 to March 27, 2018–19
								March 28 to May 17, 2020	March 28 to May 17, 2018–19
Mekaoui et al. (53)	Morocco (MIC)	No	5,342	Children <16 yrs.	ED	ED	Before after	March 16 to April 15, 2020	March 16 to April 15, 2019
Memeo et al. (54)	Italy (HIC)	Yes	1,380	Children 0-17 yrs.	Trauma ED *n* = 2	Trauma	Before after	February 23 to April 15, 2020	February 23 to April 15, 2019
Molina Gutiérrez et al. (55)	Spain (HIC)	No	6,493	Children <18 yrs.	ED	ED, trauma, poisoning	Before after	March 14 to April 17, 2020	March 14 to April 17, 2019
Montalva et al. (49)	France (HIC)	No	108	Children	Surgery	Appendicitis	Before after	January 20 to March 16, 2020	March 17 to May 11, 2020
Nabian et al. (56)	Iran (MIC)	No	877	Children <18 yrs.	Trauma ED	Trauma	Before after	March 1 to April 15, 2020	March 1 to April 15, 2018-19
Nelson et al. (57)	USA (HIC)	No	94	Children	Surgery	Testicular torsion	Before after	March 1 to May 31, 2020	January 1, 2018, to February 29, 2020
Nolen et al. (58)	USA (HIC)	No	NA	Children <3 yrs.	ED	RSV, ARI	Time series analysis	January 1 to May 31, 2020	January 1 to May 31, 2009-19
Nourazari et al. (59)	USA (HIC)	Yes	501,369	Children + adults	ED *n* = 12	ED	Before after	January 1 to August 9, 2020	January 1 to September 9, 2019
Okonkwo et al. (60)	UK (HIC)	No	3,826	Children	Surgery	emergency surgery	Before after	March 23 to May 25, 2020	March 23 to May 25, 2019
Palladino et al. (61)	Italy (HIC)	No	57	Children 4–14 years	ED	ED, seizures	Before after	March 9 to May 4, 2020	March 9 to May 4, 2019
Park et al. (62)	UK (HIC)	No	39	Children + adults	ED	Trauma	Before after	March 17 to April 15, 2020	March 17 to April 15, 2019
Peiro-Garcia et al. (63)	Spain (HIC)	No	NA	Children	Orthopedic surgery	Orthopedic surgery & Trauma	Before after	March 14 to April 14, 2020	March 14 to April 14, 2018–19
Pines et al. (64)	USA (HIC)	Yes	383,033	Children + adults	ED *n* = 144	ED, asthma, influenzae, viral infection, DKA, Appendicitis, Intussepstion, testicular torsion	Before after	March 13 to June 30, 2020	March 13 to June 30, 2019
Place (65)	USA (HIC)	No	160	Children <18 yrs.	ED	Appendicitis	Before after	March 16 to June 7, 2020	March 16 to June 7, 2019
Qasim et al. (66)	USA (HIC)	Yes	336	Children + adults	ED *n* = 2	Trauma	Before after	March 9 to April 19, 2020	March 9 to April 19, 2019
Raitio et al. (67)	Finland (HIC)	Yes	1,755	Children	Surgery *n* = 5	Trauma	Before after	March 1 to May 31, 2020	March 1 to May 31, 2017–19
Raman et al. (68)	India (MIC)	No	1,070	Children	ED	ED, PICU	Before after	April 1 to July 31, 2020	April 1 to July 31, 2019
Pediatric Surgery Trainee Research Network	UK (HIC)	Yes	87	Children	Surgery *n* = 10	Pyloric stenosis	Before after	March 23 to May 31, 2020	March 23 to May 31, 2019
Scaramuzza A	Italy (HIC)	Yes	3,912	Children <15 yrs.	ED *n* = 2	ED	Before after	February 20 to March 30, 2020	February 20 to March 30, 2019
Bun et al. (43)	Japan (HIC)	Yes	10,481	Children <15 yrs.	ED *n* = 67	Asthma	Interrupted time-series analysis	July 1, 2019, to June 30, 2020	July 1, 2018, to June 30, 2019
Sheridan et al. (69)	Ireland (HIC)	No	423	Children	ED	Trauma	Before after	March 13 to April 13, 2020	March 13 to April 13, 2009–19
Sherman et al. (70)	USA (HIC)	No	224	Children + adults	ED	Trauma	Before after	March 20 to May 14, 2020	March 20 to May 14, 2017–19
Shi et al. (42)	China (MIC)	Yes	4,877	Children	Cardiac surgery *n* = 13	Cardiac surgery	Before after	January 23 to April 8, 2020	January 23 to April 8, 2018–19
Smarrazzo A	Italy (HIC)	No		Children	ED	ED	Before after	March 1 to May 31, 2020	March 1 to May 31, 2019
Sperotto et al. (71)	Italy (HIC)	Yes	1,001	Children	PICU *n* = 4	PICU, Seizures, Surgical, Trauma, Respiratory lower tract	Before after	February 24 to April 20, 2020	December 30, 2019, to February 24, 2020
									February 24 to April 20, 2019
Williams et al. (72)	Scotland (HIC)	Yes	ED 462,437	Children 0–14 yrs.	ED n=NA, PICU *n* = 2	ED, PICU, Surgical ED, Bronchiolitis, Asthma, Seizures	Before after	March 23 to August 9, 2020	March 23 to August 9, 2016–19
			PICU 413					February 2 to August 9, 2020	February 2 to August 9, 2016–19
								March 23 to June 30, 2020	March 23 to June 30, 2016–19
Trenholme et al. (17)	New Zealand (HIC)	No	5,248	Children <2 yrs.	Hospital	LRTI, RSV, Influenza A, B, Rhinovirus/enterovirus, adenovirus	Interrupted time-series analysis	March 1 to August 31, 2020	March 1 to August 31, 2015–019
Turgut et al. (73)	Turkey (MIC)	No	2,216	Children <16 yrs.	Hospital	Orthopedic surgery & Trauma	Before-after	March 16 to May 22, 2020	March 16 to May 22, 2018 & 2019
Valitutti et al. (74)	Italy (HIC)	Yes	38,501	Children	ED *n* = 2	ED, trauma, burn, seizure	Before-after	March 1 to May 31, 2020	March 1 to May 31, 2019
Vásquez-Hoyos (58)	Colombia, Bolivia, Chile, Uruguay (MIC)	Yes	3,041	Children <18 yrs.	PICU	PICU, LRTI, bronchiolitis, RSV, influenza	Before-after	January 1 to August 31, 2020	January 1 to August 31, 2018-19
Velayos et al. (75)	Spain (HIC)	No	66	Children <18 yrs.	Surgery	Appendicitis	Before-after	January 1 to March 14, 2020	March 15 to April 30, 2020
Vierucci et al. (76)	Italy (HIC)	No	1,418	Children	ED	ED, LRTI, Trauma, Febrile Seizure, Appendicitis	Before-after	March 9 to May 31, 2020	January 1 to March 8, 2020
								March 1 to May 31, 2020	January 1 to February 29, 2019
Wei et al. (3)	China (MIC)	No	4,527	Children	Surgery	Urgent surgery	Before after	January 23 to May 21, 2020	January 23 to May 21, 2019
Wong et al. (67)	Australia (HIC)	No	621	Children	Orthopedics ED	Trauma	Before after	March 16 to April 26, 2020	March 18 to April 28, 2019
Zampieri et al. (77)	Italy (HIC)	No	95	Children	Surgery	Appendicitis	Before-after	March 1 to April 30, 2020	March 1 to April 30, 2011-19

*LIC, low-income country; MIC, middle-income country; HIC, High-income country.

### Study Settings and Data Sources

Among all the included studies, 59 were studies performed in PEDs, five in PICUs, 17 used institutional surgical databases (including three studies on congenital heart disease and one on neurosurgery), and three used a hospital-based administrative database.

### Impact on Pediatric Emergency and Intensive Care Unit Admission

#### Admissions to Pediatric Emergency Department

We included 39 studies from 19 countries including 15 multicenter studies (11, 12, 16, 18, 20–26, 28, 30, 35, 37–41, 44, 46–48, 52, 53, 55, 59, 61, 64, 65, 68, 72, 74, 76, 78–82) (Supplementary Table 1). All the included studies found a decrease in daily PED admissions during the SDM period with a median reduction of 65% [IQR: 52–72%] compared to historical cohorts. When focusing on studies including the largest number of patients (i.e., more than 200 mean daily admissions in the control period) (12, 20, 24, 35, 64, 65, 74), the reduction in PED attendance ranged from 38 to 77%.

Of these studies, 15 presented data on hospital admission following PED attendance (22, 23, 28, 35, 41, 52, 53, 55, 65, 68, 72, 74, 76, 79, 80) (Supplementary Table 2). Overall, during the SDM period, we observed a significant increase in the proportion of patients admitted to the hospital after PED visits with median ORs ranging from 1.12 to 3.51 (*n* = 13/15).

Ten studies looked at the proportions of *very-urgent* triage code on the presentation at PEDs (11, 22, 23, 25, 35, 41, 53, 59, 72, 74) but results were not consistent (Supplementary Table 3). In particular, compared with the same period in previous years, four studies found a significant increase in the proportion of *very-urgent* triage code (35, 41, 59, 74) whereas others found no difference (11, 22, 23, 53, 72) or even a decrease (25). However, when comparing to the period just before the SDM period, three studies found a significant increase in these urgent PED admissions (11, 22, 35).

### Pediatric Intensive Care Unit Admissions

We included eight studies with data regarding PICU admission (13, 26, 48, 58, 71, 81–83) with two international studies and six studies, respectively from Brazil, India, Italy, Malta, Scotland, and the USA including data from a total of 51 PICUs. All the studies found a reduction in daily PICU attendances with a median reduction of 54% [IQR: 48–65%] compared with controlled periods (Table 2). Two large multicenter studies from South America including 15 and 22 PICUs found, respectively a decrease of 53% of all the PICU admissions (13) and 83% of respiratory PICU admissions (58). Only one out of all the 51 study sites included in these studies from France found an increase of 2% in the mean number of daily admissions (48). Of note, one study from India found an overall decrease of PICU admissions of 26% in the SDM period but a significantly higher proportion of patients admitted to PICU after PED attendance (20%) compared with the control period (9, 6%) (OR: 2.35, 95%CI: 1.61–3.42) (81).

**Table 2:** PICU Admissions.

**Study**			**Study periods**								**Comparison (absolute reduction or OR (IQR)**
	**SDM period**			**Control period**			**Mean daily admission**		
**References**	**Country & region**	**Setting**	**Period**	**Number of patients**	**Age of cohort**	**Period**	**Number of patients**	**Age of cohort**	**SDM period**	**Control period**	
Araujo et al. (13)	Brazil	PICU *n* = 15	March 1 to May 31, 2020	1181	4.3 (+/-6.9)	March 1 to May 31, 2017–2019	7,473	NA	12.98	27.37	−53%
						March 1 to May 31, 2019	2,564	2.5 (+/-3.7) 2019		28.18	−54%
						March 1 to May 31, 2018	2,599	2.6 (+/-6.7) 2018		28.56	−55%
						March 1 to May 31, 2017	2,310	2.8 (+/-3.8) 2017		25.38	−49%
Degiorgio	Malta	PICU *n* = 1	March 1 to May 9, 2020	3	NA	March 1–May 9, 2019	8	NA	0.04	0.12	−63%
Graciano AL	USA	PICU *n* = 1	March 1 to 31 May, 2020	101	NA	March 1 to 31 May, 2019	195	NA	1.11	2.14	−48%
						March 1 to 31 May, 2018	275			3.02	−63%
						March 1 to 31 May, 2017	309			3.40	−67%
						March 1 to 31 May, 2016	299			3.29	−66%
						March 1 to 31 May, 2015	308			3.38	−67%
Lee L	USA/Singapore/ Australia/France	ED *n* = 5	Singapore, May 4, 2020	NA	NA	Singapore, March 15, 2020	NA	NA	NA	NA	−38 %
			Paris, May 4, 2020			Paris, March 15, 2020	NA	NA		NA	+2%
			Boston, May 4, 2020			Boston, March 15, 2020	NA	NA		NA	−12 %
			Seattle, May 4, 2020			Seattle, March 15, 2020	NA	NA		NA	−36 %
			Melbourne, May 4, 2020			Melbourne, March 15, 2020	NA	NA		NA	−34 %
Raman et al. (68)	India	ED *n* = 1	April 1 to July 31, 2020	280	NA	April 1 to July 31, 2019	790	NA	0.46	0.63	−26%
									56/280 (20%)	76/790 (9.6%)	2.35 (1.61, 3.42), *p* <0.001
Sperotto et al. (71)	Italy	PICU *n* = 4	February 24 to April 20, 2020	166	NA	December 30, 2019, to February 24, 2020	277	NA	2.96	4.95	−40%
						February 24 to April 20, 2019	259			4.71	−36%
Williams e tal. (72)	Scotland	PICU *n* = 2	March 23 to June 30, 2020	413 total	NA	March 23 to June 30, 2016-2019	413 total	NA	413 total	NA	0.52 (0.37–0.70), *p* <0.001
Vásquez-Hoyos P	Colombia, Bolivia, Chile, Uruguay	PICU 22	January 1 to August 31, 2020	234	NA	January 1 to August 31, 2018–2019	2,807	NA	0.96	5.80	−83%

*OR, Odds Ratio*.

### Impacts on Each Etiology of Illness

#### Respiratory Illness

##### Acute Respiratory Illness

Seven studies presented data regarding acute respiratory illnesses including 5 focusing on lower respiratory tract infections (LRTI) (17, 23, 26, 58, 76, 83, 84) reported from Malta, Ireland, Italy, New Zealand and four South American countries (Colombia, Bolivia, Chile, and Uruguay) (Supplementary Table 4). There was a significant decline reported in acute respiratory illness and LRTI in the five studies, all of which examined data in the PED (17, 23, 26, 76, 84) during the SDM period compared with the control periods (median decrease of 63% [50–80%]). A study from the US found that the proportion of admissions related to respiratory illness was 3.6% of all the PED admissions during the SDM period compared with 22.8% during the same period during the 10 previous years (*p* < 0.001) (84). The two studies performed in PICU also found a marked reduction in PICU admissions due to LRTI during the SDM period (52 and 83%) (58, 83).

#### Bronchiolitis

Seven studies presented data regarding acute bronchiolitis admissions (13, 23, 32, 58, 71, 82, 85) from Brazil, Australia, Ireland, USA, Scotland and four South American countries (Colombia, Bolivia, Chile, and Uruguay) (Supplementary Table 5). Three studies were performed in PEDs and found a significant reduction in hospital admissions during the SDM period ranging from 34 to 85% (23, 32, 85). The other studies looked at the data in PICU and found a similar significant reduction in children requiring critical care admission for acute bronchiolitis with a median decrease of 73% [69–78%] (13, 58, 71, 82).

#### Asthma

Seven studies presented data regarding pediatric asthma admissions (13, 43, 65, 71, 86, 87) in Brazil, the USA, the UK, Slovenia, Japan, and Scotland. Four studies performed in PED found a decrease in admissions due to asthma from 32 to 90% (43, 65, 86, 87) and two studies performed in PICUs found similar findings with a decrease of 58 to 100% (13, 82) (Supplementary Table 6).

#### Viral Infections

Seven studies (14, 17, 23, 32, 58, 65, 84) showed data regarding viral infections in Australia, Ireland, Japan, USA, New Zealand, and four South American countries (Colombia, Bolivia, Chile, and Uruguay) (Supplementary Table 7), found a significant decrease in the number of admissions due to viral infection (23, 65).

Four studies (17, 32, 58, 84) included data on RSV infections requiring PED or PICU admission during the SDM period, all of which presented a significant reduction in the incidence of presentation with RSV infections in PED (17, 32, 84) as well as in PICU (58). Similarly, PED and PICU presentations for Influenza infection reduced dramatically during the SDM period in three studies (17, 58, 65) and more mildly in a Japanese study (14). On the contrary, a study performed in New Zealand found that hospitalizations for Adenovirus or Rhinovirus/Enterovirus infections during the SDM period stayed at a similar level to the previous years (17).

#### Injuries (Trauma, Burns and Poisoning)

##### Trauma

We included 32 studies with data regarding trauma admissions to PED (*n* = 29) (15, 16, 19–21, 23, 27, 29, 37, 40, 41, 44, 51, 52, 54, 56, 59, 62, 63, 65–67, 69, 70, 73, 74, 76, 88, 89), PICU (*n* = 2) (82, 83), and a study specifically looking at neurosurgery for head trauma in children (*n* = 1) (50) (Supplementary Table 8). Of the studies in the PED, 93% of the studies (27/29) found a significant decline in admissions linked to trauma with SDMs (median reduction of 48% [35–63%]). On the contrary, 7% (2/29) reported an increase in trauma admission (16, 40, 50).

The two studies including data from patients admitted to PICU found a decrease of trauma admissions from 17 to 61% (82, 83) during the SDM period.

##### Burns

We included five studies with data regarding burns (16, 44, 74, 78, 90) respectively from the UK, Italy, and Israel (Supplementary Table 9). Four studies (44, 74, 78, 90) reported a decrease in the number of burns presenting to the PED (from 22 to 66%); while one found a slight increase in the number of burns (16) during the SDM period. However, the severity of burns at the time of hospital presentation was higher for the SDM cohorts compared to the historical (44, 78, 90). A study from the UK (78) reported that more patients presented with burns with greater total body surface area (TBSA); 50% of the patients with >5% TBSA burns in the COVID-19 pandemic compared with 5% in the control period. The study in Israel reported that the majority of burns were scalded during the SDM period; while, during previous years, causes of burns were more diverse, including scald, contact, fire, sun, and chemical injuries (44).

##### Poisoning

We included three studies with data regarding poisoning (16, 23, 59) respectively from Italy, Ireland, and Spain (Supplementary Table 10). Two of them (16, 23) did not find a significant change in the number of PED presentations for poisoning; while one found a 76% decrease in the number of hospital admissions (59). One study differentiated accidental from deliberate poisoning (23) with no differences found in both series. An Italian study found an increased incidence ratio for hospitalization in intoxicated children during the SDM period (9.0 (0.5 to 167.2), *p* = 0.14).

#### Diabetic Ketoacidosis

We found 4 studies with data regarding diabetic ketoacidosis (DKA) (31, 47, 65, 91) each from the UK, Germany, Australia, and the USA (Supplementary Table 11). Three of them found an overall increase in the referral of DKA to ED compared with previous years (93 to 264% increase). These studies also found an increase in admission of severe DKA; and the initial pH, bicarbonate and glucose levels at hospital presentations were significantly worse in the SDM group compared to the historical cohort (31, 47).

#### Surgery

##### Unplanned Surgery

A total of 20 studies met the inclusion criteria looking at unplanned surgeries, in Australia, China, France, Indonesia, Ireland, Italy, Japan, Scotland, the UK, and the USA (14, 23, 33, 36, 45, 49, 52, 57, 60, 65, 66, 68, 75–77, 82, 83, 92–95) (Supplementary Table 12). These examined data on PED/PICU admissions for acute surgical conditions, appendicitis, intussusception, testicular torsion, and pyloric stenosis as well as data on emergency surgeries performed.

Regarding studies including data on acute surgical disease, two studies found a decrease in the number of admission for acute surgical disease to PED and PICU from 9 to 40% although non-significant in the PICU (23, 83). Regarding the number of emergency surgeries performed for acute conditions, most studies found a decrease from 25 to 70% in the SDM period (77, 92, 93).

Four studies found an increase in the number of admissions with acute appendicitis during the SDM period ranging from 18 to 64% (33, 36, 45, 68). Two studies also addressed the significant increase in the proportion of perforated appendicitis (33, 68). On the contrary, three studies found a decrease in the number of admissions for appendicitis from 16 to 71% (49, 65, 76).

In addition, two studies found an increase in the number of testicular torsions (60, 65), one study found an increase in the number of pyloric stenosis (94) and one study found a decrease in the number of intussusception (65) during the SDM period compared with control periods.

##### Neurosurgery and Cardiac Surgery

Three studies reported the data of incidence of cardiac surgery for congenital heart disease during the SDM period in China, Italy, and Turkey (34, 42, 96). All three found a decrease in mean daily cases of cardiac surgery performed during the SDM period with a reduction of 52 to 88%.

Regarding studies on pediatric neurosurgery, only one study was included (50). The study reported an increase in the number of hospital admission in a pediatric neurosurgical unit during the SDM period by 14.7%.

## Discussions

Since the beginning of the COVID-19 pandemic, especially during the first wave, several modes of SDMs have been issued in most countries and regions, including school closures and restrictions on children's social activities (97). The COVID-19 pandemic was the first global pandemic in modern society with such a great impact on contemporary medical infrastructures. Therefore, facing the threat to healthcare systems capacities, SDMs were widely imposed worldwide as emergency measures and their consequences on other diseases than COVID-19 had never been described. Since then, the effects of SDM on the epidemiological dynamics of patients have been examined in various countries and regions. This systematic review summarized these studies and integrated the knowledge on the effect of SDM on acute illness in children to prepare for potential future pandemic threats during which the question of implementing SDM might arise again.

We found that SDMs in both developed and developing countries brought down the incidence of PED visits while the severity of illness at the time of the visit increased. Regarding the number of PED visits, this phenomenon might have occurred due to the decrease of morbidity or due to the avoidance of hospital visits associated with the current pandemic. The increased severity of patients might have resulted from the fact that parents tended to wait to present to the PED when their children got sick instead of immediate visits. Public communication and outreach are warranted to encourage parents/guardians to seek appropriate medical attention for emergencies of their children (28).

Likewise, a decrease in PICU admissions was also observed in almost all the reports. These results suggested that the reduction of the incidence of seasonal viral infections, and the resulting decline in the number of patients with respiratory diseases, one of the most common causes of admission to PICU, were the possible major factors behind. There was nearly a 50% decrease in general in the number of PICU admissions, and this can be important preliminary information for securing hospital beds in the event of a future pandemic or disaster, for instance, as a consequence of SDMs. These results have been confirmed by other studies published more recently (98–100).

In this study, we also compiled information on poisoning, trauma, and burns. Despite the isolation of children at home and its associated consequences both on mental health and on the access to potential accidental poisons, none of the included studies found a significant increase in accidental or deliberate poisoning. However, with the repetition of SDMs and the duration of the pandemic, the mental health of children and teenagers was strongly impacted and many studies were published since then reporting an increased incidence of mental health disorder during the COVID-19 pandemic (101, 102) but this does not seem to result in intentional poisoning increase (103).

As for burns, the number of cases decreased, but the severity at the time of consultation increased. It was encouraging that pandemic with SDMs did not translate into an increase of pediatric burn; however, parents might have chosen to seek advice from other services such as local pharmacies to avoid visits to the PED, which might explain the severity of burn at a hospital visit. The causes were more likely to be scald which might be explained by the increase in time spent at home. As for traumatic injuries, there was a general downward trend, and we believe that this was largely due to the eviction from school and the drop-in outdoor activities of kids.

DKA tended to increase, as well as the severity of the disease at the time of the hospital visit. We could assume that anxiety about presenting to the hospital led to a delay in diagnosis or receiving treatment before getting a critical condition. There has also been speculation that COVID-19 infection itself could trigger the development of DKA via direct damage to pancreatic beta cells, which might increase its actual incidence (104).

Lastly, regarding surgeries, the incidence of acute appendicitis requiring surgical intervention also increased during the SDM period, and the rate of perforated cases was reported to have risen. This might also be a result of the fact that the patients refrained from seeking medical attention until the last minute of the condition. On the contrary, the number of planned surgeries decreased significantly due to the organizational changes in the SDMs. Since a large proportion of pediatric surgical conditions, including congenital heart disease, cannot be performed on a standby basis, this might have had a significant impact on subsequent patient outcomes.

This study had limitations. First, different policies and timings of implementation must have been taken by the countries or regions in SDMs (105) although the measures were overall more strict (including national lockdowns) and similar during the first wave than during subsequent ones. For example, during the first wave, regulations and their compelling force differed from province to province and country to country. For example, in Canada, regulations for wearing masks in schools and indoors were established relatively quickly, but outdoor measures were delayed in some provinces. In some countries, such as Japan, there were no governmental regulations with strong compelling force issued as Canada, but only regulations based on a request basis to the public. Second, the magnitude of the wave of the pandemic varied from country to country, and the size of the effect of SDM itself may have been affected by this. Furthermore, since the seasons are different in the northern and southern hemispheres during the first wave, the impact of SDM on infection, respiratory diseases, and social activities may have been different in each country. Lastly, the historical cohorts to be compared in each study could be heterogeneous, and we believe that we should be cautious in evaluating them.

In conclusion, this review found that acute pediatric hospital care was significantly affected by the first wave of the COVID-19 pandemic and related SDMs in a wide range of ways. Overall admissions to PED and PICU significantly decreased. Some diseases, such as infectious diseases, decreased, while others increased in incidence and severity such as DKA. The continual effort and research in the field should be essential for us to better comprehend the effects of this new phenomenon of SDMs, to aim at minimizing possible collateral damage caused by such delays in emergency healthcare utilization, which eventually leads to protecting the well-being of children. These lessons we learned here should count for options for structuring SDMs if needed in response to future local outbreaks or pandemics in our modern society, especially given the potential harm of SDM on children's well-being (106).

## Data Availability Statement

The original contributions presented in the study are included in the article/Supplementary Material, further inquiries can be directed to the corresponding author/s.

## Author Contributions

AK and ML conceptualized, designed the review and carried out a systematic review and subsequent analysis, drafted the initial manuscript (method and result part), revised the initial manuscript, and approved the final manuscript as submitted. AK also constructed a search formula and searched. VL assessed all the titles and abstracts that remained in disagreement between the two investigators AK and ML. CS performed the statistical analyses. VL, CS, and PJ reviewed and revised the manuscript, and approved the final version as submitted. All authors contributed to the article and approved the submitted version.

## Conflict of Interest

The authors declare that the research was conducted in the absence of any commercial or financial relationships that could be construed as a potential conflict of interest.

## Publisher's Note

All claims expressed in this article are solely those of the authors and do not necessarily represent those of their affiliated organizations, or those of the publisher, the editors and the reviewers. Any product that may be evaluated in this article, or claim that may be made by its manufacturer, is not guaranteed or endorsed by the publisher.

## Appendix 1

Each digital database was searched with the following search terms.

i) *Population*: “pediatrics”, “infant, newborn”, “child”, “adolescent”, “infant” and “newborn”.
ii) *Exposures*: “social distancing”, “measure”, “policy”, “regulation”, “quarantine”, “isolation”, “non-pharmacological interventions” combined with “COVID-19”, “Novel Coronavirus” or “SARS-COV2”.
iii) *Outcomes*: “Hospitalization”, “admission”, “incidence”, “occurrence”.

The search strategy used MeSH terms unless indicated otherwise.
