# Supplementary material for: Consequences of Social Distancing Measures During the COVID-19 Pandemic First Wave on the Epidemiology of Children Admitted to Pediatric Emergency Departments and Pediatric Intensive Care Units: A Systematic Review
Source: Front Pediatr. 2022 Jun 3;10:874045. doi: 10.3389/fped.2022.874045 (PMC9204064; doi:10.3389/fped.2022.874045)
Supplement: Supplementary file 1 [file Table_1.DOCX]

**Supplemental Table 1 Admissions to Pediatric Emergency Department**

| Study | | | Study periods | | | | | | Comparison | | |
| --- | --- | --- | --- | --- | --- | --- | --- | --- | --- | --- | --- |
|  |  |  | **SDM period** | | | **Control period** | | |  |  |  |
| 1st Author | **Country** | **Setting** | **Period** | **Number of patients** | **Age of cohort** | **Period** | **Number of patients** | **Age of cohort** | **Mean daily visits (SD when available)** | **Mean daily visits (SD when available)** | **% Of reduction** |
| Akuaake LM | South Africa | ED n=1 | March 27 to April 30, 2020 | 592 | <13 yrs. | February 21 to March 26, 2020 | 3938 | <13 yrs. | **17.4** | **39.5** | -56% |
|  |  |  |  |  |  | March 27 to April 30, 2019 |  |  |  | **41.6** | -58% |
|  |  |  |  |  |  | March 27 to April 30, 2018 |  |  |  | **34.8** | -50% |
| Angoulvant F | France | ED n=6 | March 17 to April 19, 2020 | 871,543 (total) | NA | February 15 to March 16, 2020 | 871543 (total) | NA | **202.4** | **675.3** | -67% |
| Bressan S | Italy | ED n=1 | March 8 to April 20, 2020 | 796 | NA | 8 March–20 April 2019 | 2,917 | NA | **18.5** | **7.1** | -73% |
| Chelo D | Cameroon | HA n=1 | March 1 to May 31, 2020 | 1,701 | NA | March 1 to May 31, 2019 | 2284 | NA | **18.7** | **25.1** | -26% |
|  |  |  |  |  |  | March 1 to May 31, 2018 | 2159 |  |  | **23.7** | -21% |
|  |  |  |  |  |  | March 1 to May 31, 2017 | 2212 |  |  | **24.3** | -23% |
|  |  |  |  |  |  | March 1 to May 31, 2016 | 2287 |  |  | **25.1** | -26% |
|  |  |  | April 2020 |  |  | April 2019 |  |  | **18.2** | **24.9** | -27% |
|  |  |  | May 2020 |  |  | May 2019 |  |  | **14.4** | **27.1** | -47% |
| Ciofi degli Atti | Italy | ED n=1 | March 11 to April 20, 2020 | 3,265 | NA | January 1 to February 19, 2020 | 11956 | NA | **79.6 (10.4) *** | **239.1 (28.4) *** | -67% |
|  |  |  |  |  |  | February 20 to March 10, 2020 | 3604 |  |  | **180.2 (39.2) *** | -56% |
| Claudet I | France | ED n=1 | March 17 to April 19, 2020 | 2,015 | NA | March 17 to April 19, 2017-2019 | 14969 | NA | **61.1** | **151.2** | -60% |
| Clavenna A | Italy | ED n=1 | January 1 to March 31, 2020 | 2,992 | 5.8 (4.9) / 4 (2-9) | January 1 to March 31, 2019 | 4106 | 5.5 (4.7) / 4 (2-9) | **33.2** | **46.1** | -27% |
|  |  |  | February 24 to March 31, 2020 | 286 | 6.1 (5.3) / 4 (2-10) | January 1 to Feb 23,2020 | 2706 | 5.7 (4.9) / 4 (2-9) | **7.9** | **51.1** | -89% |
| D'asta | UK | ED n=1 | March 23 to April 30, 2020 | 2,936 | NA | March 23 to April 30, 2019 | 7127 | NA | **77.3** | **187.6** | -60% |
| Dann | Ireland | ED n=1 | March 1 to April 30, 2020 | 4,434 | NA | March 1 to April 30, 2019 | 9133 | NA | **73.9** | **152.2** | -49% |
|  |  |  |  |  |  | March 1 to April 30, 2018 | 8199 |  |  | **136.7** | -54% |
| Davico C | Italy | ED n=2 | January 6 - April 19, 2020 | 14,239 | <14 yrs. | January 7 to April 21, 2019 | 23016 | <14 yrs | **136.9** | **221.3** | -38% |
|  |  |  | February 24 to April 19 2020 | 3,395 | <14 yrs | February 25 to April 21 2019 | 12128 | <14 yrs | **61.7** | **220.5** | -72% |
| Dean P | USA | ED n=1 | December 31, 2019, to May 14, 2020 | 28,534 | 6.1 (1.9-13.0) | December 31 to May 14, 2015-2019 | 181824 | 6.0 (1.8-12.7) | **211.4** | **147.8** | -84% |
| Degiorgio | Malta | ED n=1 | March 1 to May 9, 2020 | 241 | < 15 yrs | March 1 to May 9, 2019 | 696 | < 15 yrs | **3.5** | **10.1** | -65% |
| Dopfer C | Germany | ED n=1 | January 1 to April 19, 2020 | 5,424 total | 5.8 (0.3) | January 1 to April 19, 2019 | 5424 total | 5.8 yrs (0.2) | **9.7 (1) *** | **26.8 (1.5) *** | -64% |
| Ferrero F | Argentina | ED n=1 | January 1 to May 31, 2020 | NA | <18 yrs | January 1 to May 31, 2019 | NA | <18 yrs | **NA** | **NA** | NA |
|  |  |  | March 2020 | NA |  | March 2019 | NA |  | **NA** | **NA** | -38% |
|  |  |  | April 2020 | NA |  | April 2019 | NA |  | **NA** | **NA** | -77% |
|  |  |  | May 2020 | NA |  | May 2019 | NA |  | **NA** | **NA** | -89% |
| Goldman RD | Canada | ED n=18 | March 17 to April 30, 2020 | 7,535 | 5 (1-11) | March 17 to April 30, 2019 | 22 654 | 5 (2–11) | **167.4 (40.7) *** | **503.4 (50.2) *** | -67% |
|  |  |  |  |  |  | December 1, 2019, to January 27, 2020 | 31 525 | 5 (2–10) |  | **543.5 (59) *** | -69% |
|  |  |  |  |  |  | January 28 to March 16, 2020 | 26 654 | 6 (2–11) |  | **544.0 (63.8) *** | -69% |
| Hampton M | UK | ED n=1 | March 24 to 7 April, 2020 | 873 | NA | March 24 to 7 April, 2019 | 2,449 | NA | **62.4** | **174.9** | -64% |
|  |  |  |  |  |  | March 10 to March 23, 2020 | 1,843 |  |  | **141.8** | -56% |
| Hartnett K | USA | ED n=3552 | March 29 to April 25, 2020 | >1,600,000 total | <10 yrs | March 31 to April 27, 2019 | >1,600,000 total | <10 yrs | **NA** | **NA** | -72% |
|  |  |  |  |  | 11-14 yrs |  |  | 11-14 yrs | **NA** | **NA** | -71% |
| Hughes H | UK | ED n=109 | March 12 to April 26, 2020 | 8,915 total | 0-14 yrs | March 14 to April 28, 2019 | 8,915 total | 0-14 yrs | **10.5** | **21.2** | −51% |
|  |  |  |  |  |  |  |  |  | **23.7** | **51.5** | −54% |
|  |  |  |  |  |  |  |  |  | **23.9** | **67.2** | −65% |
| Iozzi L | Italy | ED n=1 | March 10 to May 3, 2020 | 646 | NA | March 10 to May 3, 2019 | 2310 | NA | **12.0** | **42.8** | -72% |
| Isba R | UK/USA | ED n=2 | Weeks 13–20, 2020 | 3,269 | 0-16 yrs | Weeks 13–20, 2019 |  | 0-16 yrs | **-** |  |  |
|  |  |  | ED 1 |  |  | ED 1 | 7767 |  | **66.7** | **158.5** | -58% |
|  |  |  | ED 2 |  |  | ED 2 | 5190 |  | **33.6** | **105.9** | -68% |
|  |  |  |  |  |  | Weeks 1-12, 2020 |  |  |  |  |  |
|  |  |  |  |  |  | ED 1 | 10,402 |  |  | **123.8** | -69% |
|  |  |  |  |  |  | ED 2 | 8,361 |  |  | **99.5** | -80% |
| Rose K | UK | ED n=1 | March 21 to April 26, 2020 | 452 | 2 (1 – 6) | March 21 to April 26, 2019 | 4238 | 4 (1 – 9) | **12.6** | **117.7** | -89% |
| Kruchevsky D | Israel | ED n=1 | March 14 and April 20, 2020 | 1,134 | NA | March 14 to April 20, 2017–2019 | 2385.7 | NA | **30.6** | 64.5 | -52% |
| Kuitunen I | Finland | ED n=2 | March 16 to April 12, 2020 | 303 | NA | February 17 to March 15, 2020 | 871 | NA | **-** |  |  |
|  |  |  | ED 1 |  |  | ED 1 |  |  | **7 (3) *** | **19 (7) *** | -63% |
|  |  |  | ED 2 |  |  | ED 2 |  |  | **2.5 (3) *** | **9 (5) *** | -72% |
| Lalarukh | UK | ED n=1 | March 1 to May 31, 2020 | 355 | <18 yrs | March 1 to May 31, 2019 | 865 | <18 yrs | **3.9** | **9.5** | -59% |
| Lawrence C | Australia | ED n=1 | March 1 to May 31, 2020 | 4799 CPM | <18 yrs | March 1 to May 31, 2019 | 6550 | <18 yrs | **52.7** | **72.0** | -27% |
| Lee L | USA/Singapore/Australia/France | ED n=5 | April 2020 | NA | <18 yrs | April 2017-2019 | NA | <18 yrs | - | **-** |  |
|  |  |  | Singapore |  |  |  |  |  | **NA** | **NA** | -50% |
|  |  |  | Paris |  |  |  |  |  | **NA** | **NA** | -73% |
|  |  |  | Boston |  |  |  |  |  | **NA** | **NA** | -66% |
|  |  |  | Seattle |  |  |  |  |  | **NA** | **NA** | -59% |
| Manzoni P | Italy | ED n=2 | March 1 to April 30, 2020 | 226 | NA | March 1 to April 30, 2019 | 1428 | NA | **3.7*** | **23.4** | -84% |
| McDonnell T | Ireland | ED n=5 | February 29 to March 12, 2020 | 21,545 | NA | February 29 to March 12, 2018-2019 | 39,772 | NA | **370*** | **396*** | -7% |
|  |  |  | March 13 to March 27, 2020 |  |  | March 13 to March 27, 2018-2019 |  |  | **232*** | **435*** | -47% |
|  |  |  | March 28 to May 17, 2020 |  |  | March 28 to May 17, 2018-2019 |  |  | **195*** | **435*** | -55% |
| Mekaoui N | Morocco | ED n=1 | March 16 to April 15, 2020 | 1110 | <16 yrs | March 16 to April 15, 2019 | 4232 | <16 yrs | **37.0** | **141.1** | -74% |
| Molina Gutiérrez MA | Spain | ED n=1 | March 14 to April 17, 2020 | 1666 | <18yrs | March 14 to April 17, 2019 | 4813 | <18yrs | **49.0** | **141.6** | -65% |
| Nourazari S | USA | ED n=12 | January 1 to August 9, 2020 | 514 | 0-14 yrs | January 1 to September 9, 2019 | 1032 | 0- 14 yrs | **0.6** | **1.3** | -62% |
|  |  |  |  |  |  |  |  |  | **1.8** | **2.8** | -44% |
| Palladino F | Italy | ED n=1 | March 9 to May 4, 2020 | 3968 | 0-14 yrs | March 9, 2019, to May 4, 2019 | 16923 | 0- 14 yrs | **70.9** | **302.2** | -77% |
| Pines JM | USA | ED n=147 | March 13 to June 30, 2020 | 111,764 | <18yrs | March 13 to June 30, 2019 | 271,269 | <18yrs | **1025.4** | **2488.7** | −59% |
| Place R | USA | ED n=1 | March 16 to June 7, 2020 | NA | <18yrs | March 16 to June 7, 2019 | NA | <18yrs | **65*** | **144*** | -55% |
| Raman R | India | ED n=1 | April 1 to July 31, 2020 | 280 | NA | April 1 to July 31, 2019 | 790 | NA | **2.3** | **6.5** | -65% |
| Scaramuzza A | Italy | ED n=2 | February 20 to March 30, 2020 | 954 | <15 yrs | February 20 to March 30, 2019 | 2958 | <15 yrs | **17.0** | **10.9** | −62% |
|  |  |  |  |  |  |  |  |  | **7.4** | **31.8** | −76% |
| Valitutti F | Italy | ED n=2 | March 1 to May 31, 2020 | 9,133 | 5.9 (4.2) | March 1 to May 31, 2019 | 29,368 | 5.4 (4.7) | **100.4** | **322.7** | -69% |
| Vierucci F | Italy | ED n=1 | March 9 to May 31, 2020 | 224 | 3.5 (1.4–7.3) | January 1 to March 8, 2020 | 1194 | 3.0 (1.0–7.7) | **2.7** | **17.8** | -85% |
| Williams T | Scotland | ED n=NA | March 23 to August 9, 2020 | 462 437 total | 0–14 yrs | March 23 to August 9, 2016-2019 | 462 437 total | 0–14 yrs | **NA** | **NA** | OR 0,49 |

*The values were noted in the manuscript
