# Supplementary material for: Consequences of Social Distancing Measures During the COVID-19 Pandemic First Wave on the Epidemiology of Children Admitted to Pediatric Emergency Departments and Pediatric Intensive Care Units: A Systematic Review
Source: Front Pediatr. 2022 Jun 3;10:874045. doi: 10.3389/fped.2022.874045 (PMC9204064; doi:10.3389/fped.2022.874045)
Supplement: Supplementary file 2 [file Table_2.DOCX]

**Supplemental Table 2 Hospital admission following PED attendance**

| Reference | | | Study periods | | Proportion of admission after PED attendance | | | |
| --- | --- | --- | --- | --- | --- | --- | --- | --- |
| 1st Author | Country | Setting | SDM period | Control period | Admitted during SDM period | Admitted during control period | odds ratio | p value |
| Clavenna A | Italy | ED n=1 | January 1 to March 31, 2020 | January 1 to March 31, 2019 | 279/2992 (9.3%) | 383/4106 (9.3%) | 1.00 (0.85, 1.18) | p=0.997 |
|  |  |  | February 24 to March 31, 2020 | January 1 to Feb 23,2020 | 66/286 (23.1%) | 213/2706 (9.3%) | 3.51 (2.58, 4.78) | p<0.001 |
| Dann | Ireland | ED n=1 | March 1 to April 30, 2020 | March 1 to April 30, 2019 | 374 (8.4%) | 630 (6.9%) | 1.24 (1.08, 1.41) | p=0.002 |
|  |  |  |  | March 1 to April 30, 2018 |  | 681 (8.3%) | 1.01 (0.89, 1.16) | P=0.847 |
| Dopfer C | Germany | ED n=1 | January 1 to April 19, 2020, vs 2019 | January 1 to April 19, 2019 | 26.6 (3%) | 13.9 (1.6%) | 2.24# | p<0.001 |
| Goldman RD | Canada | ED n=18 | March 17 to April 30, 2020 | March 17 to April 30, 2019 | 535/7535 (7%) | 893/22 654 (4%) | 1.86 (1.67-2.08) | p<0.001 |
|  |  |  |  | December 1, 2019, to January 27, 2020 |  | 1319/31 525 (4%) | 1.75 (1.58-1.94) | p<0.001 |
|  |  |  |  | January 28 to March 16, 2020 |  | 1098/26 654 (4%) | 1.78 (1.60-1.98) | p<0.001 |
| Isba R | UK/USA | ED n=2 | ED 1 Weeks 13–20, 2020 | ED 1 Weeks 1-12, 2020 | 1007/3269 (30.8%) | 2598/10402 (25.0%) | 1.36 (1.17-1.58) * | p<0.001 |
|  |  |  |  | Weeks 1-12, 2019 |  | 3273/11541 (28.4%) | 1.12 (1.03- 1.22) | p=0.008 |
|  |  |  |  | Weeks 13–20, 2019 |  | 2053/7767 (26.4%) | 1.26 (1.08–1.46) * | p<0.001 |
|  |  |  | ED 2 Weeks 13–20, 2020 | ED 2 Weeks 1-12, 2020 | 293/1644 (17.8% | 895/8361 (10.7%) | 1.87 (1.51–2.32) * | p<0.001 |
|  |  |  |  | Weeks 1-12, 2019 |  | 993/8110 (12.2%) | 1.55 (1.35-1.79) | p<0.001 |
|  |  |  |  | Weeks 13–20, 2019 |  | 650/5193 (12.5%) | 1.6 (1.31–1.98) * | p<0.001 |
| Rose K | UK | ED n=1 | March 21 to April 26, 2020 | March 21 to April 26, 2019 | 127/452 (28.0%) | 631/4238 (14.9%) | 2.23 (1.79-2.79) | p<0.001 |
| Kuitunen I | Finland | ED n=2 | ED 1 March 16 to April 12, 2020 | ED 1 February 17 to March 15, 2020 | 16/211 (7.6%) | 29/605 (4.7%) | 1.63 (0.87-3.06) | p=0.13 |
|  |  |  | ED 2 March 16 to April 12, 2020 | ED 2 February 17 to March 15, 2020 | 40/92 (44%) | 100/266 (38%) | 1.28 (0.79-2.07) | p=0.32 |
| Manzoni P | Italy | ED n=2 | March 1 to April 30, 2020 | March 1 to April 30, 2019 | 0.3 (8.8%) | 1.2 (5.0%) | 1.78 (1.15-2.76) * | p=0.02 |
| McDonnell T | Ireland | ED n=5 | February 29 to March 12, 2020 | February 29 to March 12, 2018-2019 | 54/370 (15%) | 60/396 (14%) | 0.96 (0.64, 1.43) | p=0.829 |
|  |  |  | March 13 to March 27, 2020 | March 13 to March 27, 2018-2019 | 34/232 (15%) | 64/435 (15%) | 1.00 (0.63, 1.56) | p=0.984 |
|  |  |  | March 28 to May 17, 2020 | March 28 to May 17, 2018-2019 | 29/195 (15%) | 62/435 (14%) | 1.05 (0.65, 1.69) | p=0.838 |
| Mekaoui N | Morocco | ED n=1 | March 16 to April 15, 2020 | March 16 to April 15, 2019 | 471/1110 (42.4%) | 811/4232 (19.2%) | 3.11 (2.70, 3.58) | p<0.005 |
| Pines JM | USA | ED n=147 | March 13 to June 30, 2020 | March 13 to June 30, 2019 | 7795/111764 (7%) | 13708/271269 (5%) | 1.43 (1.37, 1.45) | p<0.001 |
| Place R | USA | ED n=1 | March 16 to June 7, 2020 | March 16 to June 7, 2019 | 10.7 (16,4%) | 15.2 (10.5%) | 1.67# | p=0.07 |
| Scaramuzza A | Italy | ED n=2 | ED 1 February 20 to March 30, 2020 | ED 1 February 20 to March 30, 2019 | 50/664 (7.5%) | 42/1749 (2.4%) | 3.31 (2.17, 5.04) | p<0.001 |
|  |  |  | ED 2 February 20 to March 30, 2020 | ED 2 February 20 to March 30, 2019 | 40/290 (13.8%) | 98/1209 (8.1% | 1.81 (1.23, 2.69) | p=0.003 |
| Valitutti F | Italy | ED n=2 | March 1 to May 31, 2020 | March 1 to May 31, 2019 | 1455/9133 (15.93%) | 2812/29368 (9.57%) | 1.79 (1.67, 1.92) | p<0.001 |
| Vierucci F | Italy | ED n=1 | March 9 to May 31, 2020 | January 1 to March 8, 2020 | 91/224 (40.6%) | 235/1194 (19.7%) | 2.79 (2.06, 3.78) | p<0.001 |

*Values were noted in the manuscript

# CI could not be calculated
