# Supplementary material for: Consequences of Social Distancing Measures During the COVID-19 Pandemic First Wave on the Epidemiology of Children Admitted to Pediatric Emergency Departments and Pediatric Intensive Care Units: A Systematic Review
Source: Front Pediatr. 2022 Jun 3;10:874045. doi: 10.3389/fped.2022.874045 (PMC9204064; doi:10.3389/fped.2022.874045)
Supplement: Supplementary file 3 [file Table_3.DOCX]

**Supplemental Table 3 Very Urgent triage code on the presentation at PEDs**

| Reference | | | Study periods | | The proportion of very urgent triage category^#^ PED attendance | | | |
| --- | --- | --- | --- | --- | --- | --- | --- | --- |
|  |  |  |  |  |  |  |  |  |
| 1st Author | Country | Setting | SDM period | Control period | Admitted during SDM period | Admitted during control period | odds ratio | p-value |
| Akuaake LM | South Africa | ED n=1 | March 27 to April 30, 2020 | February 21 to March 26, 2020 | 25/592 (4.2%) | 30/1342 (2.2%) | 1.93 (1.12, 3.31) | p=0.015 |
|  |  |  |  | March 27 to April 30, 2019 |  | 40/1413 (2.8%) | 1.51 (0.91, 2.52) | p=0.108 |
|  |  |  |  | March 27 to April 30, 2018 |  | 41/1183 (3.5%) | 1.23 (0.74, 2.04) | p=0.427 |
| Clavenna A | Italy | ED n=1 | January 1 to March 31, 2020 | January 1 to March 31, 2019 | 229/2992 (7.6%) | 306/4106 (7.5%) | 1.03 (0.86, 1.23) | p=0.769 |
|  |  |  | February 24 to March 31, 2020 | January 1 to Feb 23,2020 | 43/286 (14.6%) | 186/2706 (6.8%) | 2.40 (1.68, 3.43) | p<0.001 |
| Dann | Ireland | ED n=1 | March 1 to April 30, 2020 | March 1 to April 30, 2019 | 19/4434 (0.4%) | 23/9133 (0.25%) | 1.70 (0.93-3.13) | p=0.082 |
|  |  |  |  | March 1 to April 30, 2018 |  | 26/8199 (0.31%) | 1.35 (0.75-2.45) | p=0.316 |
| Dean P | USA | ED n=1 | December 31, 2019, to May 14, 2020 | December 31, to May 14, 2015-2019 | 951/28534 (3.3%) | 7254/181824 (4.0%) | 0.83 (0.77-0.89) | p<0.001 |
| Goldman RD | Canada | ED n=18 | March 17 to April 30, 2020 | March 17 to April 30, 2019 | 1488/7535 (20%) | 3571/22 654 (16%) | 1.31 (1.23-1.41) | p<0.001 |
|  |  |  |  | December 1, 2019, to January 27, 2020 |  | 5747/31 525 (18%) | 1.10 (1.04-1.18) | p=0.002 |
|  |  |  |  | January 28 to March 16, 2020 |  | 4781/26 654 (18%) | 1.13 (1.06-1.20) | p<0.001 |
| Rose K | UK | ED n=1 | March 21 to April 26, 2020 | March 21 to April 26, 2019 | 94/452 (20.8%) | 616/4238 (14.5%) | 6,26 (2.60-10.4) * | p<0.001 |
| McDonnell T | Ireland | ED n=5 | February 29 to March 12, 2020 | February 29 to March 12, 2018-2019 | 78/370 (21%) | 80/396 (19%) | 1.02 (0.72, 1.45) | p=0.904 |
|  |  |  | March 13 to March 27, 2020 | March 13 to March 27, 2018-2019 | 47/232 (20%) | 88/435 (20%) | 1.00 (0.67, 1.49) | p=0.993 |
|  |  |  | March 28 to May 17, 2020 | March 28 to May 17, 2018-2019 | 38/195 (19%) | 87/435 (20%) | 0.97 (0.63, 1.48) | p=0.881 |
| Molina Gutiérrez MA | Spain | ED n=1 | March 14 to April 17, 2020 | March 14 to April 17, 2019 | 665/1666 (39,9%) | 1608/4813 (33,4%) | 1.32 (1.18, 1.49) | p<0.001 |
| Scaramuzza A | Italy | ED n=2 | ED 1 February 20 to March 30, 2020 | ED 1 February 20 to March 30, 2019 | 32/664 (4.8%) | 24/1749 (1.3%) | 3.64 (2.13-6.23) | p<0.001 |
|  |  |  | ED 2 February 20 to March 30, 2020 | ED 2 February 20 to March 30, 2019 | 4/290 (1.4%) | 6/1209 (0.5%) | 2.80 (0.79-10.00) | p=0.209 |
| Valitutti F | Italy | ED n=2 | March 1 to May 31, 2020 | March 1 to May 31, 2019 | 46/9133 (0.50%) | 34/29368 (0.11%) | 4.37 (2.80-6.81) | p<0.001 |

*The values were noted in the manuscript

# Very urgent triage code included patients that either required immediate resuscitation or had life-threatening conditions that require prompt evaluation by the emergency team
