# Supplementary material for: Consequences of Social Distancing Measures During the COVID-19 Pandemic First Wave on the Epidemiology of Children Admitted to Pediatric Emergency Departments and Pediatric Intensive Care Units: A Systematic Review
Source: Front Pediatr. 2022 Jun 3;10:874045. doi: 10.3389/fped.2022.874045 (PMC9204064; doi:10.3389/fped.2022.874045)
Supplement: Supplementary file 4 [file Table_4.DOCX]

**Supplemental Table 4 Impact on Respiratory Illness**

| Reference | | | Disease | SDM period | Control period | Number of admissions | | | | Difference with  control period | Odds ratio for respiratory illness  among all PED admission |
| --- | --- | --- | --- | --- | --- | --- | --- | --- | --- | --- | --- |
|  |  |  |  |  |  | **SDM period** | | **Control period** | |  |  |
| 1st Author | **Country** | **Setting** | **Type of disease** | **Period** | **Period** | **Absolute number$** | **Mean daily admission** | **Absolute number$** | **Mean daily admission** |  |  |
| Degiorgio | Malta | ED n=1 | Respiratory illnesses | March 1 to May 9, 2020 | March 1 to May 9, 2019 | 84/266 (31.6%) | 1.22 | 346/729 (47.4%) | 5.01 | -76% |  |
| Dann | Ireland | ED n=1 | Respiratory illnesses | March 1 to April 30, 2020 | March 1 to April 30, 2019 | 864 | 14.40 | 1742 | 29.03 | -50% |  |
|  |  |  |  |  | March 1 to April 30, 2018 |  |  | 1742 | 29.03 | -50% |  |
| Nolen L | USA | HA n=1 | ARI | January 1 to May 31, 2020 | January 1 to May 31, 2009-2019 | 194.5 per 1000 | NA | 224.8 per 1000 | NA | -13% |  |
| Sperotto F | Italy | PICU n=4 | LRTI | February 24 to April 20, 2020 | December 30, 2019, to February 24, 2020 | 14 | 0.25 | 82 | 1.46 | -83% |  |
|  |  |  |  |  | February 24 to April 20, 2019 |  |  | 29 | 0.52 | -52% |  |
| Trenholme A | New Zealand | ED n=1 | LRTI | March 1 to August 31, 2020 | March 1 to August 31, 2015-2019 | 159 | 0.87 | 5089 | 5.56 | -84% |  |
| Vásquez-Hoyos P | Colombia, Bolivia, Chile, Uruguay | PICU n=22 | LRTI | January 1 to August 31, 2020 | January 1 to August 31, 2018-2019 | 234 | 0.96 | 2807 | 2.32 | -58% |  |
| Vierucci F | Italy | ED n=1 | LRTI | March 9 to May 31, 2020 | January 1 to March 8, 2020 | 34/224 (15.2%) | 0.41 | 501/1194 (42%) | 7.48 | -95% | 0.25 (0.17, 0.36) p<0.001 |
