# Supplementary material for: Consequences of Social Distancing Measures During the COVID-19 Pandemic First Wave on the Epidemiology of Children Admitted to Pediatric Emergency Departments and Pediatric Intensive Care Units: A Systematic Review
Source: Front Pediatr. 2022 Jun 3;10:874045. doi: 10.3389/fped.2022.874045 (PMC9204064; doi:10.3389/fped.2022.874045)
Supplement: Supplementary file 5 [file Table_5.DOCX]

**Supplemental Table 5 Impacts on Bronchiolitis**

| Reference | | | SDM period | Control period | Number of Admissions | | | | Difference with  control period | ORs for bronchiolitis  among all PED admission |
| --- | --- | --- | --- | --- | --- | --- | --- | --- | --- | --- |
|  |  |  |  |  | **SDM period** | | **Control period** | |  |  |
| 1st Author | **Country & Region** | **Setting** | **Period** | **Period** | **Absolute number$** | **Mean daily admission** | **Absolute number$** | **Mean daily admission** |  |  |
| Araujo OR | Brazil | PICU n=15 | March 1 to May 31, 2020 | March 1 to May 31, 2019 | 147/1181 (12.4%) | 2.45 | 834/2564 (32.5%) | 9.16 | -73% |  |
|  |  |  |  | March 1 to May 31, 2018 |  |  | 842/2599 (32.4%) | 9.25 | -74% |  |
|  |  |  |  | March 1 to May 31, 2017 |  |  | 729/2310 (31.6%) | 8.01 | -69% |  |
| Britton P | Australia | ED n=1 | April 1 to June 30, 2020, | Jan 1, 2015, to March 30, 2020, | NA | NA | NA | NA | 70.8% Lower than predicted |  |
| Dann | Ireland | ED n=1 | March 1 to April 30, 2020 | March 1 to April 30, 2019 | "81/4434 (1.8%)" | 1.35 | 144/9133 (1.6%) | 2.4 | -44% | 1.16 (0.88, 1.53) p=0.285 |
|  |  |  |  | March 1 to April 30, 2018 |  |  | 123/8199 (1.5%) | 2.05 | -34% | 1.22 (0.92, 1.62) p=0.165 |
| Friedrich F | Brazil | ED n=NA | March 1 to June 30, 2020 | March 1 to June 30, 2019 | 682 $ | NA | 4,526.90 $ | NA | -85% |  |
|  |  |  |  | March 1 to June 30, 2018 |  |  | 3,909.50 $ | NA | -83% |  |
|  |  |  |  | March 1 to June 30, 2017 |  |  | 3,872.30 $ | NA | -82% |  |
|  |  |  |  | March 1 to June 30, 2016 |  |  | 3,048.10 $ | NA | -78% |  |
| Graciano AL | USA | PICU n=1 | March 1 to May 31, 2020 | March 1 to May 31, 2019 | 8/101 (7.9%) | 0.09 | 29/195 (14.9%) | 0.32 | -72% | 0.49 (0.22, 1.12) p=0.086 |
|  |  |  |  | March 1 to May 31, 2018 |  |  | 31/275 (11.2%) | 0.34 | -74% | 0.68 (0.30, 1.53) p=0.345 |
|  |  |  |  | March 1 to May 31, 2017 |  |  | 39/309 (12.6%) | 0.43 | -79% | 0.60 (0.27, 1.32) p=0.198 |
|  |  |  |  | March 1 to May 31, 2016 |  |  | 24/299 (8.0%) | 0.26 | -66% | 0.99 (0.43, 2.27) p=0.973 |
|  |  |  |  | March 1 to May 31, 2015 |  |  | 26/308 (8.4%) | 0.29 | -69% | 0.93 (0.41, 2.13) p=0.869 |
| Williams T | Scotland | PICU n=2 | March 23 to June 30, 2020 | March 23 to June 30, 2016-2019 | 0 | 0 | 39 | 0.10 | -100% |  |
| Vásquez-Hoyos P | Colombia, Bolivia, Chile, Uruguay | PICU n=22 | January 1 to August 31, 2020 | January 1 to August 31, 2018-2019 | 126 | 0.52 | 1504 | 3.11 | -83% |  |

$hospital /100,000 children, OR; Odds Ratio
