# Supplementary material for: Consequences of Social Distancing Measures During the COVID-19 Pandemic First Wave on the Epidemiology of Children Admitted to Pediatric Emergency Departments and Pediatric Intensive Care Units: A Systematic Review
Source: Front Pediatr. 2022 Jun 3;10:874045. doi: 10.3389/fped.2022.874045 (PMC9204064; doi:10.3389/fped.2022.874045)
Supplement: Supplementary file 6 [file Table_6.DOCX]

**Supplemental Table 6 Impacts on Asthma**

| Reference | | | SDM period | Control period | Number of Admissions | | | | Difference with  control period | ORs for asthma  among all PED admission |
| --- | --- | --- | --- | --- | --- | --- | --- | --- | --- | --- |
|  |  |  |  |  | **SDM period** | | **Control period** | |  |  |
| 1st Author | **Country** | **Setting** | **Period** | **Period** | **Absolute number$** | **Mean daily admission** | **Absolute number$** | **Mean daily admission** |  |  |
| Araujo OR | Brazil | PICU n=15 | March 1 to May 31, 2020 | March 1 to May 31, 2019 | 101/1181 (8.6%) | 1.11 | 306/2564 (11.9%) | 3.36 | -67% |  |
|  |  |  |  | March 1 to May 31, 2018 |  |  | 321/2599 (12.4%) | 3.53 | -69% |  |
|  |  |  |  | March 1 to May 31, 2017 |  |  | 242/2310 (10.5%) | 2.66 | -58% |  |
| Chavasse R | UK | ED n=1 | Week 12 to 19, 2020 | Week 8 to 11, 2020 | 2(0-5) * | 0.29 | 17 (16-19) * | 2.43 | -88% |  |
|  |  |  |  | Week 12 to 19, 2017-2019 |  |  | NA |  | -90% |  |
| Graciano AL | USA | PICU n=1 | March 1 to May 31, 2020 | March 1 to May 31, 2019 | 8/101 (7.9%) | 0.09 | 29/195 (14.9%) | 0.32 | -72% | 0.49 (0.22, 1.12) p=0.086 |
|  |  |  |  | March 1 to May 31, 2018 |  |  | 31/275 (11.2%) | 0.34 | -74% | 0.68 (0.30, 1.53) p=0.345 |
|  |  |  |  | March 1 to May 31, 2017 |  |  | 39/309 (12.6%) | 0.43 | -79% | 0.60 (0.27, 1.32) p=0.198 |
|  |  |  |  | March 1 to May 31, 2016 |  |  | 24/299 (8.0%) | 0.26 | -66% | 0.99 (0.43, 2.27) p=0.973 |
|  |  |  |  | March 1 to May 31, 2015 |  |  | 26/308 (8.4%) | 0.29 | -69% | 0.93 (0.41, 2.13) p=0.869 |
| Krivec U | Slovenia | ED n=1 | March 16 to April 20, 2020 | March 16 to April 20, 2019 | 2 | 0.06 | 9 | 0.26 | -78% |  |
|  |  |  |  | March 16 to April 20, 2018 |  |  | 9 | 0.26 | -78% |  |
|  |  |  |  | March 16 to April 20, 2017 |  |  | 7 | 0.20 | -71% |  |
| Pines JM | USA | ED n=144 | March 13 to June 30, 2020 | March 13 to June 30, 2019 | 1,329 | 12.19 | 5,512 | 50.57 | -76% |  |
| Bun S | Japan | ED n=67 | July 1, 2019, to June 30, 2020 | July 1, 2018, to June 30, 2019 | 4,236 | 11.61 | 6,245 | 17.11 | -32% |  |
| Williams T | Scotland | PICU n=2 | March 23 to June 30, 2020 | March 23 to June 30, 2016-2019 | 0 | 0 | 4 | 0.01 | -100% |  |

OR; odds ratio, *weekly admissions
