# Supplementary material for: Consequences of Social Distancing Measures During the COVID-19 Pandemic First Wave on the Epidemiology of Children Admitted to Pediatric Emergency Departments and Pediatric Intensive Care Units: A Systematic Review
Source: Front Pediatr. 2022 Jun 3;10:874045. doi: 10.3389/fped.2022.874045 (PMC9204064; doi:10.3389/fped.2022.874045)
Supplement: Supplementary file 7 [file Table_7.DOCX]

**Supplemental Table 7 Impacts on Viral Infections**

| Reference | | | Type of virus | SDM period | Control period | Number of admissions | | | | Difference with  control period | Odds ratio for infection among all PED admission |
| --- | --- | --- | --- | --- | --- | --- | --- | --- | --- | --- | --- |
|  |  |  |  |  |  | **SDM period** | | **Control period** | |  |  |
| 1st Author | **Country** | **Setting** | **Type of disease** | **Period** | **Period** | **Absolute number$** | **Mean daily admission** | **Absolute number$** | **Mean daily admission** |  |  |
| Britton P | Australia | ED n=144 | RSV | April 1 to June 30, 2020, | January 1, 2015, to March 30, 2020, | NA | NA | NA | NA | -94.3% (22.8), p=0.026* |  |
| Dann | Ireland | ED n=1 | Virally mediated illnesses | March 1 to April 30, 2020 | March and April 2019 | 1108/4434 (25%) | 18.47 | 2310/9133 (25.3%) | 74.52 | -75% | 0.98 (0.91, 1.07) p=0.702 |
|  |  |  |  |  |  |  |  | 2151/8199 (26.2%) | 69.39 | -73% | 0.94 (0.86, 1.02) p=0.127 |
| Kishimoto K | Japan | HA n=257 | Influenzae | March 1 to June 30, 2020 | July 1, 2018, to February 29, 2020 | NA | NA | NA | NA | -21 (16.5), p<0.001# |  |
| Nolen L | USA | HA n=1 | RSV | January 1 to May 31, 2020 | January 1 to May 31, 2009-2019 | 74.0 per one thousand | NA | 61.9 per 1000 | NA | 20% |  |
| Pines JM | USA | ED n=144 | Viral infection subgroup | March 13 to June 30, 2020 | March 13 to June 30, 2019 | 2,523 | 23.15 | 6,713 | 61.59 | -62% |  |
|  |  |  | Influenza subgroup |  |  | 900 | 8.26 | 5501 | 50.47 | -84% |  |
| Trenholme A | New Zealand | HA n=1 | RSV | March 1 to August 31, 2020 | March 1 to August 31, 2015-2019 | 2 | 0.01 | 1347 | 1.47 | -99% |  |
|  |  |  | Influenza A |  |  | 1 | 0.01 | 238 | 0.26 | -98% |  |
|  |  |  | Influenza B |  |  | 0 | 0.00 | 126 | 0.14 | -100% |  |
|  |  |  | Rhinovirus/enterovirus |  |  | 252 | 1.38 | 1715 | 1.87 | -27% |  |
|  |  |  | Adenovirus |  |  | 41 | 0.22 | 353 | 0.39 | -42% |  |
| Vásquez-Hoyos P | Colombia, Bolivia, Chile, Uruguay | PICU n=22 | RSV | January 1 to August 31, 2020 | January 1 to August 31, 2018-2019 | 45 | 0.19 | 1070 | 2.21 | -92% |  |
|  |  |  | Influenza |  |  | 7 | 0.03 | 65 | 0.13 | -79% |  |

OR; odds ratio, *Difference in mean frequency from expected (standard error)

#Estimated coefficient of changes of inpatients per week (standard error)
