# Supplementary material for: Consequences of Social Distancing Measures During the COVID-19 Pandemic First Wave on the Epidemiology of Children Admitted to Pediatric Emergency Departments and Pediatric Intensive Care Units: A Systematic Review
Source: Front Pediatr. 2022 Jun 3;10:874045. doi: 10.3389/fped.2022.874045 (PMC9204064; doi:10.3389/fped.2022.874045)
Supplement: Supplementary file 8 [file Table_8.DOCX]

**Supplemental Table 8 Impacts on Poisoning**

| Reference | | | Type of intoxication | SDM period | Control period | Number of admissions | | | | Difference with  control period | ORs for poisoning among all PED admission |
| --- | --- | --- | --- | --- | --- | --- | --- | --- | --- | --- | --- |
|  |  |  |  |  |  | **SDM period** | | **Control period** | |  |  |
| 1st Author | **Country** | **Setting** | **Type of disease** | **Period** | **Period** | **Absolute number$** | **Mean daily admission** | **Absolute number$** | **Mean daily admission** |  |  |
| Bressan S | Italy | ED n=1 | Poisoning | March 8 to April 20, 2020 | March 8 to April 20, 2019 | 14/796 (1.8%) | 0.33 | 11/2917 (0.4%) | 0.26 | 27% |  |
| Dann | Ireland | ED n=1 | Accidental poisoning | March 1 to April 30, 2020 | March 1 to April 30, 2019 | 28/4434 (0.63%) | 0.47 | 26/9133 (0.28%) | 0.43 | 8% | 2.23 (1.31, 3.81) p=0.003 |
|  |  |  |  |  | March 1 to April 30, 2018 |  |  | 58/8199 (0.53%) | 0.97 | -52% | 0.89 (0.57, 1.41) p=0.627 |
|  |  |  | Deliberate poisoning | March 1 to April 30, 2020 | March 1 to April 30, 2019 | 9/4434 (0.2%) | 0.15 | 10/9133 (0.1%) | 0.17 | -10% | 1.86 (0.75, 4.57) p=0.172 |
|  |  |  |  |  | March 1 to April 30, 2018 |  |  | 14/8199 (0.17%) | 0.23 | -36% | 1.19 (0.51, 2.75) p=0.685 |
| Molina Gutiérrez MA | Spain | ED n=1 | Accidental poisoning | March 14 to April 17, 2020 | March 14 to April 17, 2019 | 5/1666 (0.3%) | 0.15 | 21/4813 (0.4%) | 0.62 | -76% | 0.60 (0.23, 1.60) p=0.302 |

OR; odds ratio, *Difference in mean frequency from expected (standard error)

#Estimated coefficient of changes of inpatients per week (standard error)
