# Supplementary material for: Consequences of Social Distancing Measures During the COVID-19 Pandemic First Wave on the Epidemiology of Children Admitted to Pediatric Emergency Departments and Pediatric Intensive Care Units: A Systematic Review
Source: Front Pediatr. 2022 Jun 3;10:874045. doi: 10.3389/fped.2022.874045 (PMC9204064; doi:10.3389/fped.2022.874045)
Supplement: Supplementary file 9 [file Table_9.DOCX]

**Supplemental Table 9 Impacts on Burn**

| Reference | | | SDM period | Control period | Number of admissions | | | | | Difference with  control period | ORs for burn among all PED admission | |  |
| --- | --- | --- | --- | --- | --- | --- | --- | --- | --- | --- | --- | --- | --- |
|  |  |  |  |  | **SDM period** | | **Control period** | | |  |  |  |  |
| 1st Author | **Country** | **Setting** | **Period** | **Period** | **Absolute number** | **Mean daily admission** | **Absolute number** | **Mean daily admission** |  | | |  | |
| Bressan S | Italy | ED n=1 | March 8 to April 20, 2020 | March 8 to April 20, 2019 | 12/796 (1.5%) | 0.28 | 11/2917 (0.4%) | 0.26 | 9% | | |  | |
| D'asta F | UK | ED n=1 | March 23 to April 30, 2020 | March 23 to April 30, 2019 | 83/2936 (2.8%) | 2.18 | 107/7127 (1.5%) | 2.82 | -22% | | | 1.91 (1.43, 2.55) p<0.001 | |
| Kruchevsky D | Israel | ED n=1 | March 14 to April 20, 2020 | March 14 to April 20, 2017–2019 | 16/1134 (1.4%) | 0.43 | 21/2385.7 (0.9%) * | 0.57 | -24% | | |  | |
| Mann JA | UK | ED n=1 | March 23 to May 31, 2020 | March 23 to May 31, 2019 | 64/5031 (1.3%) | 0.93 | 83/12599 (0.7%) | 1.20 | -23% | | | 1.94 (1.40, 2.70) p<0.001 | |
| Valitutti F | Italy | ED n=2 | March 1 to May 31, 2020 | March 1 to May 31, 2019 | 56/9133 (0.6%) | 0.62 | 165/29368 (0.6%) | 1.81 | -66% | | |  | |

OR; odds ratio, *Difference in mean frequency from expected (standard error)
