# Supplementary material for: Consequences of Social Distancing Measures During the COVID-19 Pandemic First Wave on the Epidemiology of Children Admitted to Pediatric Emergency Departments and Pediatric Intensive Care Units: A Systematic Review
Source: Front Pediatr. 2022 Jun 3;10:874045. doi: 10.3389/fped.2022.874045 (PMC9204064; doi:10.3389/fped.2022.874045)
Supplement: Supplementary file 11 [file Table_11.DOCX]

**Supplemental Table 11 Diabetic Ketoacidosis**

| Reference | | | Type of disease | SDM period | Control period | DKA | | | | | | Severe DKA | | | | | |
| --- | --- | --- | --- | --- | --- | --- | --- | --- | --- | --- | --- | --- | --- | --- | --- | --- | --- |
|  |  |  |  |  |  | **SDM period** | | **Control period** | | **Difference with  control period** | **ORs for poisoning among all PED admission** | **SDM period** | | **Control period** | | **Difference with  control period** | **ORs for poisoning among all PED admission** |
| 1st Author | **Country & Region** | **Setting** | **Type of disease** | **Period** | **Period** | **Absolute number$** | **Mean daily admission** | **Absolute number$** | **Mean daily admission** |  |  | **Absolute number$** | **Mean daily admission** | **Absolute number$** | **Mean daily admission** |  |  |
| Bastemur E | UK | transportation team | DKA | March 1 to July 31, 2020 | March 1 to July 31, 2018-2019 | 31 | 0.20 | 31 | 0.10 | 100% |  | 11/31 (35%) | 0.07 | 13/31 (42%) | 0.04 | 69% |  |
|  |  |  |  |  | March 1 to July 31, 2015-2019 |  |  | 12 (11-20) * | 0.08 | 158% |  |  |  |  |  |  |  |
| Kamrath C | Germany | ED n=217 | T1D | March 13 to May 13, 2020 | March 13 to May 13, 2019 | 238/532 (44.7%) | 3.90 | 123/503 (24.5%) | 2.02 | 93% | 2.50 (1.92, 3.26) p<0.001 | 103/532 (19.4%) | 1.69 | 70/503 (13.9%) | 1.15 | 47% | 1.37 (1.04, 1.81) p=0.03 |
|  |  |  |  |  | March 13 to May 13, 2018 |  |  | 110/456 (24.1%) | 1.80 | 116% | 2.55 (1.94, 3.35) p<0.001 |  |  | 56/456 (12.3%) | 0.92 | 84% | 1.55 (1.15, 2.1) p=0.004 |
| Lawrence C | Australia | ED n=1 | T1D | March 1 to May 31, 2020 | March 1 to May 31, 2015-2019 | 8/11 (73%) | 0.09 | 11/42 (26%) | 0.02 | 264% | 7.52 (1.69, 33.50) p=0.012 | 5/11 (45%) | 0.05 | 2/42 (5%) | 0.00 | 1274% | 16.7 (2.0, 194.7 p<0.003 |
| Pines JM | USA | ED n=147 | DKA subgroup | March 13 to June 30, 2020 | March 13 to June 30, 2019 | 214 | 1.96 | 229 | 2.10 | -7% |  |  |  |  |  |  |  |

T1D; Type 1 Diabetes

OR; odds ratio,

$ % of all PED admissions if available

*Median admission per year (IQR)
