# Supplementary material for: Consequences of Social Distancing Measures During the COVID-19 Pandemic First Wave on the Epidemiology of Children Admitted to Pediatric Emergency Departments and Pediatric Intensive Care Units: A Systematic Review
Source: Front Pediatr. 2022 Jun 3;10:874045. doi: 10.3389/fped.2022.874045 (PMC9204064; doi:10.3389/fped.2022.874045)
Supplement: Supplementary file 12 [file Table_12.DOCX]

**Supplemental Table 12 Impacts on Unplanned Surgery**

| Reference | | | Type of disease | SDM period | | Control period | | Number of admissions | | | | | | Surgery performed | | | | |
| --- | --- | --- | --- | --- | --- | --- | --- | --- | --- | --- | --- | --- | --- | --- | --- | --- | --- | --- |
|  |  |  |  |  |  |  |  | **SDM period** | | **Control period** | | **Difference with control period** | **Odds ratio** | **SDM period** | | **Control period** | |  |
| 1st Author | **Country** | **Setting** | **Type of disease** | **Period** | **Age** | **Period** | **Age** | **Absolute number** | **Mean daily admission** | **Absolute number** | **Mean daily admission** |  |  | **Absolute number** | **Mean daily surgery** | **Absolute number** | **Mean daily surgery** | **Difference with control period** |
| Dann | Ireland | ED n=1 | Acute surgical disease (Appendicitis, surgical abscess, | March 1 to April 30, 2020 | children | March 1 to April 30, 2019 | children | 133/4434 (2.9%) | 2.22 | 183/9133 (2%) | 3.05 | -27% | 1.51 (1.21, 1.90), p<0.001 |  |  |  |  |  |
|  |  |  | torsion hydatid, testicular torsion) |  |  | March 1 to April 30, 2018 | children |  |  | 222/8199 (2.7%) | 3.70 | -40% | 1.11 (0.89, 1.38), p=0.343 |  |  |  |  |  |
| Fisher JC | USA | ED n=3 | Appendicitis | March 1 to May 7, 2020 | children | January 1, 2014, to June 1, 2019 | children | 55 | 0.82 | 1291 | 0.65 | 18% |  |  |  |  |  |  |
|  |  |  | Perforated appendicitis |  |  |  |  | 25/55 (45%) | 0.37 | 351/1291 (27%) | 0.18 | 106% | 2.23 (1.29, 3.85), p = 0.003 |  |  |  |  |  |
| Gerall CD | USA | Surgery n=1 | Appendicitis | March 1 to May 31, 2020 | 11.07 (8.31-14.08) | March 1, 2019, to May 31, 2019 | 13.15 (8.36-15.84) | 48 | 0.53 | 41 | 0.45 | 17% |  | 36/48 (75.0%) | 0.40 | 38/41 (92.7%) | 0.42 | -5% |
| Gunadi DR | Indonesia | Surgery n=1 | Acute surgical disease (Digestive, neonate, urology) | March 1 to May 31, 2020 | children | Mar 2019 – Feb 2020 | children |  |  |  |  |  |  | 20 | 0.22 | 103 | 0.31 | -29% |
|  |  |  |  |  |  | Dec 2019 – Feb 2020 |  |  |  |  |  |  |  |  |  | 29 | 0.47 | -53% |
| Kishimoto K | Japan | HA n=210 | Appendicitis | March 1 to June 30, 2020 | children | July 1, 2018, to February 29, 2020 | children |  |  |  |  | **-5.7 (2.5), p=0.025 $** |  |  |  |  |  |  |
| Kvasnovsky C | USA |  | Appendicitis | March 31 to May 3, 2020 | 12.4 (7.8-15.4) | March 31 to May 3, 2019 | children | 55 | 0.87 | 41 | 1.24 | 34% |  | 30/55 (55%) | 0.48 | 41/41 (100%) | 1.24 | -61% |
|  |  |  |  |  |  | March 31 to May 3, 2018 |  |  |  | 53 | 1.61 | 4% |  |  |  | 53/53 (100%) | 1.61 | -70% |
|  |  |  |  |  |  | March 31 to May 3, 2017 |  |  |  | 56 | 1.70 | -2% |  |  |  | 56/56 (100%) | 1.70 | -72% |
| Lee-Archer P | Australia | Surgery n=1 | Appendicitis | March 16 to May 5, 2020 | 11.0 (7.8-13.0) | March 16 to May 5, 2019 | 10.2 (8.4-13.3) | 48 | 0.96 | 57 | 1.14 | -16% |  |  |  |  |  |  |
| Manzoni P | Italy | ED n=2 | Acute surgical disease | March 1 to April 30, 2020 | children | March 1 to April 30, 2019 | children | NA | 0.1/3.7 (2.2%) |  | 0.1/23.4 (0.3%) | 0% | **6.65 (2.41, 18.31), p=0,002*** |  |  |  |  |  |
| Montalva L | France | ED n=1 | Appendicitis | March 17 to May 11, 2020 | 11.1 (9.1-13.2) | January 20 to March 16, 2020 | 8.9 (5.7-12.2) |  |  |  |  |  |  | 69 | 1.25 | 39 | 0.70 | 79% |
| Nelson C | USA | Surgery n=1 | Testicular torsion | March 1 to May 31, 2020 | 15.2 (14.2-16.0) | January 1, 2018, to February 29, 2020 | 14.2 (12.6-15.8) |  |  |  |  |  |  | 17 | 0.19 | 77 | 0.10 | 110% |
|  |  |  |  |  |  | March 1 to May 31, 2018-2019 |  |  |  |  |  |  |  |  |  | 15 | 0.08 | 163% |
| Okonkwo I | UK | Surgery n=1 | Emergency surgery | March 23 to May 25, 2020 | children | March 23 to May 25, 2019 | children |  |  |  |  |  |  | 878 | 13.94 | 2948 | 46.79 | -70% |
| Pines JM | USA | ED n=144 | Appendicitis subgroup | March 13 to June 30, 2020 | children | March 13 to June 30, 2019 | children | 921 | 8.45 | 1144 | 10.50 | -19% |  |  |  |  |  |  |
|  |  |  | Intussusception subgroup |  |  |  |  | 40 | 0.37 | 69 | 0.63 | -42% |  |  |  |  |  |  |
|  |  |  | Testicular torsion subgroup |  |  |  |  | 96 | 0.88 | 89 | 0.82 | 8% |  |  |  |  |  |  |
| Place R | USA | ED n=1 | Appendicitis | March 16 to June 7, 2020 | 10 (7-13) | March 16 to June 7, 2019 | 11 (9-14) | 90 | 1.08 | 70 | 0.66 | 64% |  | 82/90 (91%) | 0.99 | 70/70 (100%) | 0.66 | 50% |
|  |  |  | Perforated appendicitis |  |  |  |  | 35/90 (39%) | 0.42 | 13/70 (19%) | 0.12 | 250% | 2.79 (1.34, 5.83), p=0.005 |  |  |  |  |  |
| Paediatric Surgery Trainee Research Network | UK | Surgery n=10 | Pyloric stenosis | March 23 to May 31, 2020 | 34 (26–41) in days | March 23 to May 31, 2019 | 31 (24–41) in days |  |  |  |  |  |  | 47 | 0.68 | 40 | 0.58 | 18% |
| Sperotto F | Italy | PICU n=4 | Acute surgical disease | February 24 to April 20, 2020 | children | December 30, 2019, to February 24, 2020 | NA | 62 | 1.11 | 68 | 1.21 | -9% | **0.86 (0.63, 1.24) #** |  |  |  |  |  |
|  |  |  |  |  |  | February 24 to April 20, 2019 |  |  |  | 83 | 1.51 | -27% | **0.87 (0.62, 1.23) #** |  |  |  |  |  |
| Williams T | Scotland | ED n=NA | Acute surgical disease | March 23 to August 9, 2020 | children 0–14 yrs | March 23 to August 9, 2018-2019 | children |  |  |  |  |  | **1.03 (0.95, 1.12), p=0.46*** |  |  |  |  |  |
| Velayos M | Spain | Surgery n=1 | Appendicitis | January 1 to March 14, 2020 | 9.3+/-3.1 | March 15 to April 30, 2020 | 10.7+/-3 |  |  |  |  |  |  | 25 | 0.34 | 41 | 0.89 | -39% |
| Vierucci F | Italy | ED n=1 | Appendicitis | March 1 to May 31, 2020 | children | January 1 to February 29, 2020 | children | 5 | 0.05 | 10 | 0.17 | -71% |  |  |  |  |  |  |
| Wei Y | China | Surgery n=1 | Emergency surgery | January 23 to May 21, 2020 | children | January 23 to May 21, 2019 | children |  |  |  |  |  |  |  | 67.86 MWS |  | 90.14 MWS | -25% |
| Zampieri N | Italy | Surgery n=1 | Appendicitis | March 1 to April 30, 2020 | children | March 1 to April 30, 2011-2019 | children |  |  |  |  |  |  | 12 | 0.20 | 160.00 | 0.30 | -33% |

*Calculated by authors

$ coefficient of change in the number of patients per week between the 2 periods

# Incidence rate ratios

MWS; mean weekly surgeries
